# How do Empirical Metacommunity Ecologists (not) Define Local Communities and How Could These be Better Defined?

**DOI:** 10.1111/ele.70298

**Published:** 2025-12-28

**Authors:** Lluís Serra‐Domínguez, Otso Ovaskainen, Nerea Abrego

**Affiliations:** ^1^ Department of Biological and Environmental Science University of Jyväskylä Jyväskylä Finland

**Keywords:** agent‐based simulation, co‐occurrence, fitness influence, individual‐based network, interactions, local community, metacommunity, metacommunity ecology, metacommunity theory, modularity

## Abstract

Metacommunity Theory is among the most widely used theoretical frameworks in empirical community ecology. A central assumption of this framework is that individuals are structured into local communities, which collectively form the metacommunity. Thus, the concept of the local community is fundamental for connecting empirical observations with theoretical predictions. However, through a literature review, we show that most empirical studies conceptualised within Metacommunity Theory lack explicit spatial definitions of local communities. Among those that do, few provide ecological justification. We argue that this mismatch between theoretical assumptions and empirical practice hinders the interpretability and comparability of empirical results. To address this gap, we propose three alternative approaches for delineating local communities. These are based on whether conspecific and heterospecific individuals overlap in their space‐use, interact with each other or have reciprocal fitness effects. Using agent‐based simulations, we show how these three definitions may result in different delineations of local communities and that local communities do not necessarily form discrete units. To align empirical studies with Metacommunity Theory, we urge ecologists to explicitly define what spatial units they conceptualise as local communities. We also offer guidelines on what complementary data could be collected to achieve ecologically justified delineations of local communities.

## Graphical abstract

Metacommunity Theory assumes discrete local communities, yet empirically delineating such local communities is challenging. To resolve the gap between theoretical assumptions and empirical practice, we propose three alternative approaches for conceptualising what should be considered as a local community. Using agent‐based simulations, we demonstrate that delineating local communities based on spatiotemporal co‐occurrence leads to contrasting results compared to delineations based on species interactions.

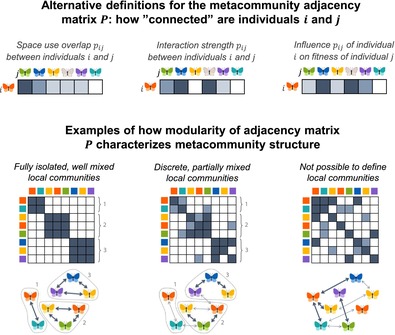

## 1 Introduction

Metacommunity Theory (MT) is one of the most widely adopted theoretical frameworks in empirical community ecology. It provides a unified and flexible approach for understanding how different ecological processes influence the dynamics of community assembly across different spatial scales (Leibold et al. 2004; Leibold and Chase 2018; Thompson et al. 2020). In MT, a metacommunity is defined as a network of local communities within which individuals interact. The local communities are linked to each other through the dispersal of individuals (Leibold et al. 2004). Thus, local communities form the building blocks of metacommunities. Despite the theoretical significance and widespread application of the concept of a local community, there is no standardised method for delineating or defining local communities in natural systems. In fact, as we show in this paper, defining local communities in real‐world empirical systems can be challenging, as local communities may lack discrete boundaries or those boundaries may be difficult to identify. This difficulty hinders the connection between empirical research and MT, as it obscures our understanding of the scale‐dependency of assembly processes.

The definition of ecological communities throughout the history of community ecology has been a controversial topic, starting from the contrasting Clementsian versus Gleasonian views in the early XX century. Clements (1916) defined ecological communities as static and delineable units, whereas Gleason (1926) acknowledged that the spatial boundaries among communities are not sharp and that their composition is dynamic over time. While community ecologists have generally leaned towards the Gleasonian perspective, the debate over the definition of ecological communities has persisted (Fauth et al. 1996; Morin 2011; Ricklefs 2008; Stroud et al. 2015). There are two main perspectives on defining an ecological community: one focuses on the spatiotemporal co‐occurrence of species and individuals (Begon et al. 1990; Mittelbach and McGill 2019; Morin 2011), while the other emphasises the importance of interactions among individuals in addition to their shared spatial use (Begon et al. 2006; Leibold and Chase 2018; Stroud et al. 2015). Although the diverse definitions of ‘ecological community’ have been acknowledged, the practical implications of these discrepancies in empirical studies have rarely been addressed.

In the context of MT, empirical studies on patchy landscapes have often assumed that every habitat patch supports a separate local community. This assumption is justified if the individuals spend most of their lifetimes and interact primarily within a single habitat patch, dispersing among the patches only occasionally. This might be the case for some groups of organisms, such as fungi inhabiting discrete deadwood units (Abrego 2022), symbionts in hosts (Mihaljevic 2012) or aquatic organisms in rockpools (De Meester et al. 2005). However, as has been remarked in single species metapopulation studies, frequent interpatch movement is not uncommon, especially in small and transient habitats (Bowne and Bowers 2004; Fahrig 2013; Harrison 1991). In such scenarios, individuals may regularly reproduce with individuals from other habitat patches, thus constituting a patchy population rather than a metapopulation (Harrison 1991; Harrison and Taylor 1997; Mayer et al. 2009; Stacey et al. 1997). Scaling up to multiple species, it follows that a local community may span multiple habitat patches if individuals frequently move and interact across habitat patches. Conversely, for dispersal limited organisms, a single large habitat patch may contain several distinct local communities occupying different parts of the patch. Without any supporting evidence, it can often remain unclear to what extent the spatial boundaries of the habitat patches match the boundaries of the local communities (Fahrig 2013).

In landscapes that have continuous rather than patchy spatial structure, individuals may form a continuum in their space use and interactions, challenging the delimitation of local communities as discrete units. The difficulty of delineating local communities in natural systems is often overlooked in empirical research by simply treating sampling units (e.g., sampling plots or sites) as local communities. While this approach is a pragmatic choice, it disconnects the interpretation of empirical results from the foundational assumptions of MT, namely, that individuals typically spend most of their lifetimes within a single local community (Leibold et al. 2004; Leibold and Chase 2018).

We argue that the variation in how local communities are (or are not) defined across studies hampers the interpretability and comparability of their results, as evidenced by the fact that the relative importance of the inferred assembly processes varies across the spatial grain and extent of the sampling (Chase and Knight 2013; Viana and Chase 2019). Importantly, when several taxonomic groups are simultaneously included in the same study, defining the spatial scale of local communities uniformly may lead to misleading conclusions about the relative importance of local vs. regional processes (e.g., macroorganisms vs. microorganisms; Astorga et al. 2012, Nemergut et al. 2013, Heino et al. 2015).

Despite the importance of local community definition in relating empirical studies to theory, how local communities are delineated in practice has received relatively little attention. The aim of this study is three‐fold. First, we review empirical studies framed within the metacommunity framework to investigate how local communities are currently defined and delineated in the ecological literature. We specifically ask to what extent local communities are explicitly defined and to what extent these definitions are compatible with the underlying assumptions of metacommunity theory. Second, we develop three alternative theoretical definitions for how local communities could be defined in empirical studies. These definitions are based on overlapping space‐use of individuals, potential for interactions among individuals and reciprocal fitness effects among individuals. To evaluate how these definitions behave under different metacommunity scenarios, we implement a virtual ecologist approach where we apply the theoretical definitions to data generated by agent‐based simulations. Third, we discuss to what extent the proposed definitions can be applied to real community data, where individual‐level interactions and dispersal events are often partially or entirely unknown. We synthesise our findings by recommending empirical metacommunity ecologists to explicitly define what they refer to as local communities and by providing guidelines on what kind of additional data may be collected for objectively delineating local communities in accordance with the conceptual definitions made.

## 2 How are Local Communities Defined in Empirical Studies?

### 2.1 Literature Review Methodology

We conducted a literature review on empirical papers framed within MT. The aim of the review was to quantify the proportion of papers that explicitly defined the local communities and the criteria they used. We searched the Web of Science September 18th, 2025, for papers that were published from 2006 to 2025 and that contained the word ‘metacommunity’ in the title. The search identified 620 papers. We only considered studies that were explicitly framed within MT and that contained empirical data (either non‐manipulative observational or manipulative experimental, excluding studies in which the local communities were created in silico). Through stratified random sampling, we selected 10 papers per year. For some years, the number of suitable papers was lower, such as 2006, which included three suitable papers and 2009, which included nine papers, resulting in a final sample of 192 papers reviewed (see Appendix S1). For each paper, we inferred how the authors conceptualised landscape structure (patchy or continuous), what spatial unit they conceptualised as a local community and to what extent they justified the choice made. Additionally, we classified each study in terms of the focal environment (freshwater, marine or terrestrial).

For the underlying landscape structure, we differentiated between studies that considered patches of suitable habitat embedded within an unhabitable matrix (hereafter patchy landscapes) and studies that considered habitat variation to be of continuous nature (continuous landscapes). We included in the latter category homogeneous landscapes, mosaics of discrete habitat types and gradients of habitat quality or resource availability (Biswas and Wagner 2012). We considered that the authors justified the conceptualisation of their landscape as patchy or continuous if they provided supporting data, literature references or if the classification appeared evident based on the study system (e.g., fishes in a pond system).

In studies conceptualised as patchy landscapes, we evaluated whether the authors considered a one‐to‐one correspondence between habitat patches and local communities or if the authors considered alternative habitat patch‐community configurations, such as scenarios where local communities span multiple habitat patches or single habitat patches contain multiple local communities. We further evaluated the extent by which the choice was supported with evidence, which we classified in three categories: (1) studies that made an implicit assumption without providing any data, references or an intuitive explanation; (2) studies that supported their patch‐community configuration with indirect species‐level evidence, such as discussing patterns of variation in community composition or using species traits as a proxy of connectivity; (3) studies where the authors provided individual level data, such as capture‐mark‐recapture data on movement patterns or direct observations about the scale at which individuals interacted.

In studies that the authors conceptualised as continuous landscapes, we assessed at what spatial level of the sampling design the local communities were defined. We classified the papers into those where local communities corresponded to the smallest level at which the data were collected (i.e., the sampling unit level), in contrast to those where pooled samples from several locations were considered to represent a local community. We then evaluated the level of support provided by the authors for their delineation of local communities, using the same criteria as for the patchy landscapes: (1) no justification, (2) indirect justification by species‐level data, (3) direct justification by individual‐level data.

Finally, we evaluated whether the studies (on both patchy and continuous landscapes) considered the boundaries of the local community to be the same for all individuals and species (disjoint communities) or whether they considered that some individuals may frequently move between local communities and/or interact with individuals from other local communities (overlapping community structures).

### 2.2 Literature Review Results

**FIGURE 1:**
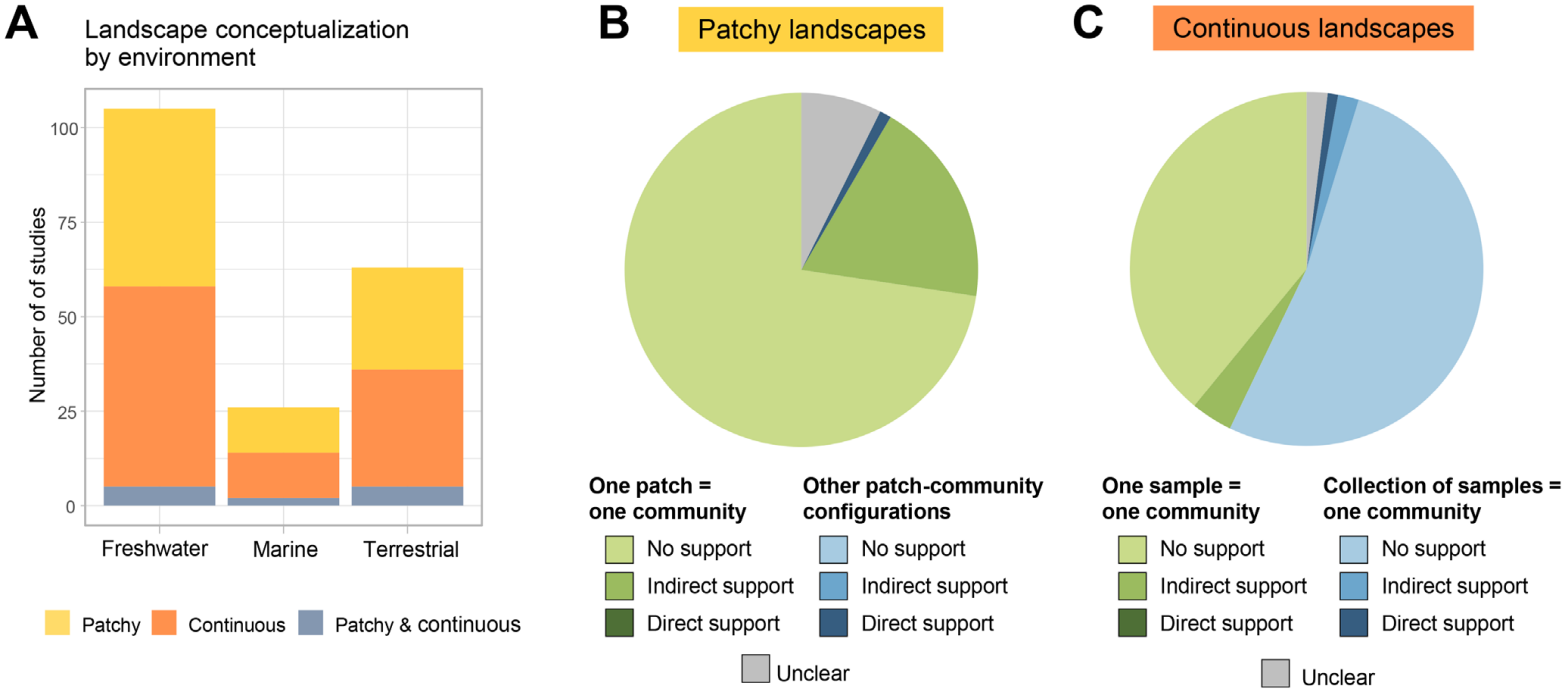
Results of literature review on empirical studies framed within Metacommunity Theory. (A) Number of studies conceptualised as patchy or continuous for each ecosystem type. (B) Among studies framed as patchy landscapes, proportion of studies that considered each patch to host a single local community (green) versus alternative patch‐community configurations (blue). (C) Among studies framed as continuous landscapes, proportion of studies that assume the finest (spatially smallest) sampling unit to equal a local community (green), versus studies that assume that collections of finest sampling units equal a local community (blue). In panels (B) and (C), darker colour shadings indicate higher levels of empirical evidence behind the conceptualizations.

Most studies were carried out in freshwater environments (54%), followed by terrestrial (32%) and marine environments (13%) (Figure 1A). Regardless of the environment, the total number of studies was balanced between patchy (45%) and continuous (50%) landscapes, with few studies (5%) considering that the landscape simultaneously included patchy and continuous components (Figure 1A). Among studies conceptualised as patchy landscapes, the vast majority (90%) provided some support for justifying why they considered the patches to be more suitable habitats than the matrix or it was evident from the landscape.

Within patchy landscapes, the vast majority (92%) considered each habitat patch to hold a single local community (Figure 1B). Only one study (1%) considered another type of patch‐community configuration (Lawson et al. 2019) and for seven studies (7%) it remained unclear at which spatial scale the local communities were conceptualised. Among studies that assumed a one‐to‐one correspondence between local communities and habitat patches, the majority (80%) did not provide any supporting justification for it. Indirect support was provided by 20% of the studies, for example, by referring to dispersal traits and/or habitat connectivity considerations (Fernandes et al. 2014; Jaworski et al. 2016). No study provided direct support. The study that considered an alternative patch‐community configuration used microsatellite genetic markers to conclude that there was either panmixia or at most five subpopulations across the nine islands, with dispersal rates varying across species (Lawson et al. 2019).

For the studies framed as continuous landscapes, 42% assumed the smallest level in the sampling design to represent a local community, whereas 56% assumed that some aggregates of sampling locations represented the local communities and in two articles (2%) it was unclear at what sampling level they considered local communities to be represented (Figure 1C). Similarly to patchy landscapes, the level of support for justifying these choices was limited: the vast majority of studies (93%) did not provide any support, 6% of the studies provided indirect support and 1 study provided direct support with genetic methods (Manier and Arnold 2006; Figure 1C).

In patchy landscapes, no studies explicitly considered the possibility that local communities may not be discrete units, but that they may overlap across habitat patches. Only a minority (14%) of the studies explicitly justified the discrete nature of local communities or it appeared evident from the study system (e.g., studies on endoparasites or obligate symbionts). In continuous landscapes, where the overlapping nature of communities could be expected to be prevalent, only two studies explicitly mentioned this possibility by acknowledging that biotic interactions may occur in a spatial continuum and that local communities may overlap (Manier and Arnold 2006; Valanko et al. 2015).

In summary, the results of the review demonstrate that empirical studies often fail to explicitly define what spatial units they conceptualise as local communities. Among those that do, few provide supporting evidence for their choice. Implicitly, empirical studies appear to define local communities based on space use and assume local communities to have discrete boundaries. The review also highlighted that while most of the theoretical advances in metacommunity ecology focus on patch‐based landscape configurations (Leibold and Chase 2018), empirical community ecologists often work with continuous landscape configurations, which do not directly align with the patch‐based theory.

To bridge the gap between theoretical and empirical metacommunity studies, we next suggest how local communities can be explicitly defined and then discuss how those definitions may be applied in empirical studies. Motivated by the broad variety of empirical metacommunity studies pointed out by the literature review, we aim to provide generally applicable definitions for what is considered a local community. In particular, the definitions of local communities should be applicable for all types of landscape structures (such as patchy or continuous); they should be applicable for all types of environments and taxonomical groups and they should not assume a priori any correspondence between habitat units and local communities.

## 3 How to Define Local Communities: Theoretical Considerations

**FIGURE 2:**
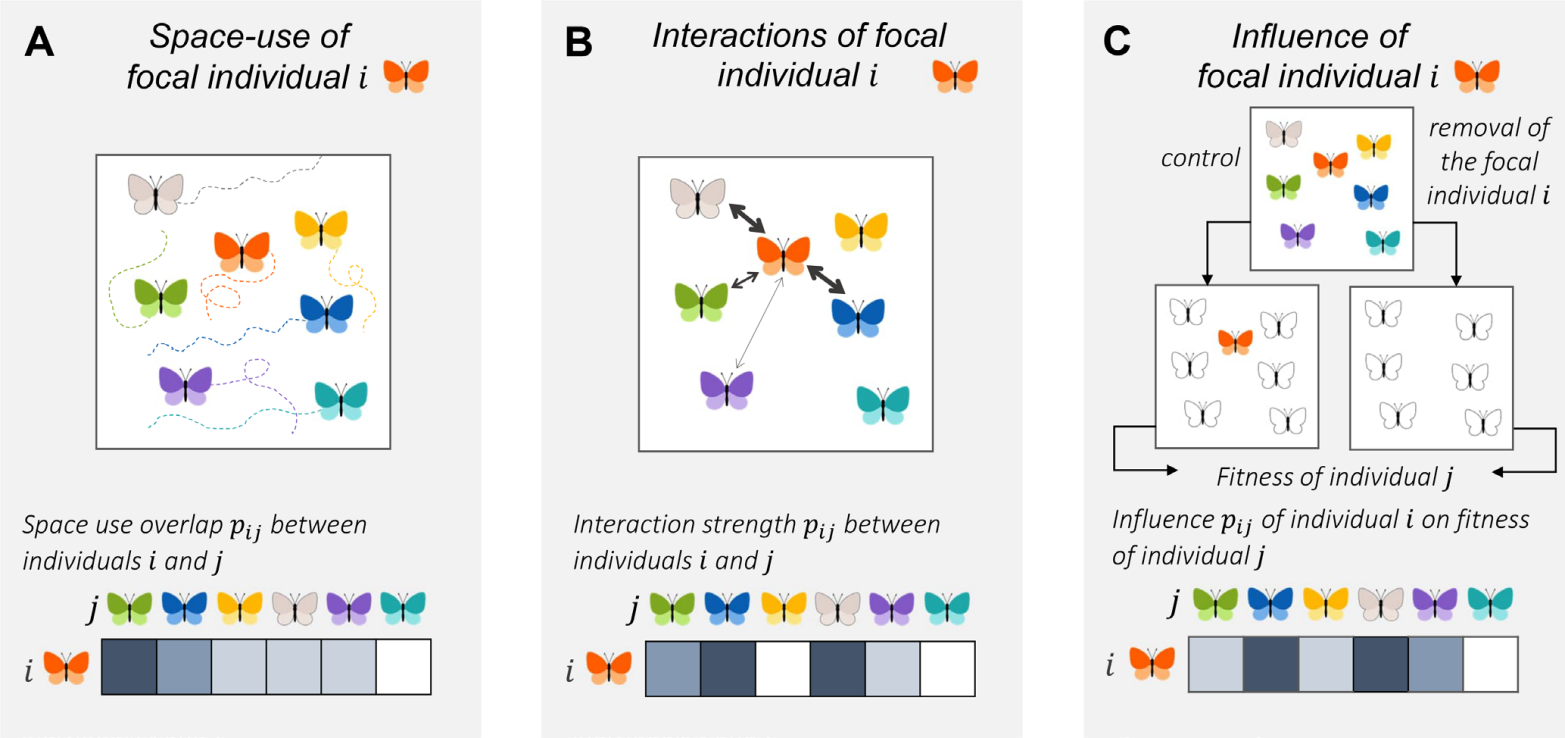
Space‐use and network–based definitions for the local community of a focal individual. The space‐based definition (A) defines the local community of a focal individual as a probability density function of the individual's location, modelled from the realised or expected space used. The interaction‐based definition (B) defines the local community of a focal individual as those individuals with whom the focal individual interacts. The influence‐based definition (C) defines the local community of a focal individual as those individuals whose fitness it influences, measured by asking how the removal of the focal individual would influence the fitness of the other individuals.

**FIGURE 3:**
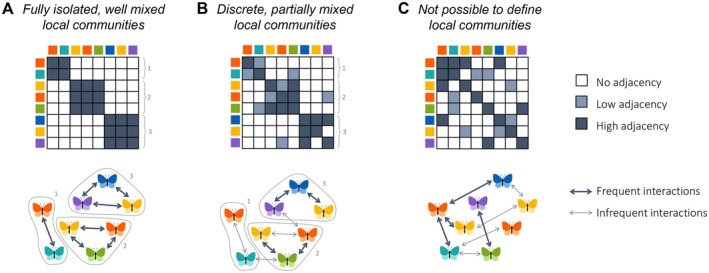
Defining local communities through modularity of the adjacency matrix. The figure illustrates three hypothetical metacommunities that have varying level of modularity in their adjacency matrices $P=\left\{p_{\mathrm{ij}}\right\}$. Darker colours in the adjacency matrix and thicker arrows in the cartoon illustration represent heavier edge weights, that is, stronger connections between individuals. Panel A represents a collection of fully isolated local communities. Panel B represents a classical metacommunity scenario, where well‐defined local communities have some level of dispersal between them. Panel C represents a metacommunity that cannot be partitioned into well‐defined disjoint local communities.

In this section we discuss how local communities may be theoretically defined and delineated, without yet considering whether or how such definitions can be applied to empirical studies, a question to which we will return in the Discussion. We define local communities through two hierarchical levels. First, we define the local community of a focal individual by determining what other individuals constitute its local community and to what degree (Figure 2). We then examine to what extent the local communities of the focal individuals combine to form well‐defined and distinct local communities, as often implied in metacommunity studies (Figure 3).

### 3.1 Local Community of a Focal Individual

When defining a focal individual's local community, we distinguish between a space‐use based approach that only considers the spatiotemporal co‐occurrence among individuals (Figure 2A) and two network‐based approaches that also consider the interactions among individuals (Figure 2B) or the influences of such interactions (Figure 2C).

#### 3.1.1 Space Use ‐Based Definition

This definition aligns with the perspective of defining an ecological community as ‘the species that occur together in space and time,’ a definition that has been repeatedly proposed (Stroud et al. 2015) and, as highlighted by the review above, is most commonly and often implicitly assumed in empirical studies.

In the space use‐based definition, we define a local community of a focal individual i as a function of space $C_{i}\left(x,y\right)$ either as a binary indicator describing which locations are $\left(C_{i}\left(x,y\right)=1\right)$ and which locations are not $\left(C_{i}\left(x,y\right)=0\right)$ part of its local community or as a continuously varying function measuring space‐use intensity at the location $\left(x,y\right)$. In both cases, we normalise $C_{i}\left(x,y\right)$ into a probability density function, so that $C_{i}\left(x,y\right)\text{dxdy}$ can be viewed to describe the probability that at any particular time the individual i is located within a small area dxdy around the location $\left(x,y\right)$. The function $C_{i}\left(x,y\right)$ measures the area in which the intra‐ and interspecific interactions of the focal individual i may take place (Casper et al. 2003; Costa‐Pereira et al. 2022), accounting for its movement scale (Nathan et al. 2012). We note that the time dimension is not included here and hence we consider $C_{i}$ as averaged over some focal time span, which may be, for example, the lifetime of the focal individual, one season or any other timescale that is considered relevant from the viewpoint of how local communities should be defined. To determine the extent of overlap in the local communities of any two individuals i and j (Figure 2A), we measure the overlap between their respective probability density functions of space use, here by computing Bhattacharya coefficient (Bhattacharyya 1943), defined as $p_{\mathrm{ij}}=\int \sqrt{C_{i}\left(x,y\right)C_{j}\left(x,y\right)}\text{dxdy}$. This index ranges from 0, no overlap in space use, to 1, identical space use.

#### 3.1.2 Network‐Based Definitions: Interactions and Influences

To consider the perspective that the definition of local communities should include the interactions among individuals (Leibold et al. 2004; Stroud et al. 2015), we propose two alternatives, either considering ecologically relevant direct interactions between individuals or considering the total influences that the individuals exert on each other.

From a network perspective, we define the local community of the focal individual i as the vector $C_{i}=\left\{p_{\mathrm{ij}}\right\}$, where $p_{\mathrm{ij}}$ denotes the extent to which an individual j belongs to the community of individual i, that is, the degree of the membership of individual j to the local community of individual i (Fletcher et al. 2013; Guimarães 2020). Therefore, unlike the space‐use definition, we define the local community of a focal individual by a collection of other individuals rather than by a spatial area. In such individual‐based network, the individuals form the nodes, which are connected to each other by edges of weight $p_{\mathrm{ij}}$. Community membership can be binary $\left(p_{\mathrm{ij}}\in \left\{0,1\right\}\right)$ or it can involve continuous variation in its weight $\left(p_{\mathrm{ij}}\in \left[0,1\right]\right)$. We next describe the two alternative ways in which the $p_{\mathrm{ij}}$ can be defined.

To account for the role of direct interactions in shaping local communities, we define $p_{\mathrm{ij}}$ based on inter‐ and intraspecific interactions among pairs of individuals (Guimarães 2020) (Figure 2B). If considering the presence or absence of an interaction, $p_{\mathrm{ij}}=1$ if the individuals i and j ever interact and $p_{\mathrm{ij}}=0$ if they do not. If considering variation in the weights of edges, $p_{\mathrm{ij}}$ can derived from the frequency at which the individuals i and j interact. We note that while this interaction‐based definition may sound intuitive, it has several caveats. First, considering only direct interactions may not capture other pathways of influence. For example, in resource competition, individuals may never directly interact, yet they affect each other by depleting the pool of available resources. Similarly, in multitrophic systems, direct interactions may not capture indirect effects such as trophic cascades. Second, accounting only for pairwise interactions may fail to capture higher order interactions, which involve more than two individuals at a time (Wootton 1994; Mayfield and Stouffer 2017). Third, not all types of interactions are equally relevant and they may vary in strength (Wootton and Emmerson 2005) or symmetry (Adler et al. 2018; Bascompte et al. 2006). It can be difficult to parameterize the interaction intensity $p_{\mathrm{ij}}$ to account for such complexities.

Motivated by the above‐mentioned limitations, we consider an alternative definition, where we focus on the influences that the individuals have on each other (Figure 2C), reflecting their requirement and impact niches (Leibold 1995; Letten et al. 2017). The influence‐based definition can be theoretically or experimentally implemented by removing the focal individual i from the community and tracking the influence of such perturbation on the surrounding individuals' fitness. We thus define the local community of a focal individual again as $C_{i}=\left\{p_{\mathrm{ij}}\right\}$, but now $p_{\mathrm{ij}}$ measures the extent to which the removal of the focal individual i influences the fitness of the individual j. Since biotic interactions can be asymmetric, $p_{\mathrm{ij}}$ is not necessarily equivalent to $p_{\mathrm{ji}}$. For example, commensalism where i benefits from j, whereas j experiences no net effect from the interaction with i, will produce $p_{\mathrm{ij}}=0$ (because removal of i does not influence j) and $p_{\mathrm{ji}}<0$ (because of removal of j reduces the fitness of i).

### 3.2 Local Community of a Collection of Individuals

We defined the local community of a focal individual i using alternative approaches to calculate $p_{\mathrm{ij}}$, which measures whether or how strongly individuals i and j are linked through spatial proximity or interactions. The metacommunity adjacency matrix P is defined as the collection of all such pairwise links, $P=\left\{p_{\mathrm{ij}}\right\}$. We next discuss how this matrix can be used to delineate local communities as collections of individuals.

In network terminology, systems composed of distinct, densely interconnected subsystems are called modular (Grilli et al. 2016; Newman and Girvan 2004). A perfectly modular network is characterised by a diagonal block structure of its adjacency matrix, where each block represents a self‐contained subsystem with no external connections. Such structure in the metacommunity adjacency matrix would represent a collection of fully isolated local communities (Figure 3A). A classical metacommunity scenario with well‐defined yet interconnected local communities corresponds to an adjacency matrix with non‐zero off‐block values while still retaining a clear diagonal block structure (Figure 3B). A significant departure from a diagonal block structure indicates that it may not be possible to define local communities as discrete and disjoint units (Figure 3C). Therefore, we argue that local communities can be defined by finding out to which extent the metacommunity adjacency matrix can be partitioned into distinct blocks.

The degree of compartmentalization of a network or its adjacency matrix's block structure can be measured by calculating its modularity (Newman and Girvan 2004), which compares the observed weight of a given division of a network (partition) to the expected weight of a random network with the same degree distribution. High modularity values, close to 1, reflect a strong diagonal block structure and well‐defined local communities, whereas values close to 0 suggest random connections. We suggest that local communities can be identified by finding the partition that maximises the modularity of the metacommunity adjacency matrix, as this will identify groups of individuals for which internal connections are stronger than connections to other groups of individuals (Fortunato 2010; Newman and Girvan 2004).

## 4 How Do Different Definitions of Local Community Relate to Each Other?

In this section we apply a virtual ecologist approach (Zurell et al. 2010) to explore how consistently the alternative definitions delineate local communities under different metacommunity scenarios. Before presenting the results, we describe the agent‐based model used to create simulated communities, how we implemented the delineation of local communities based on the three alternative definitions and how we evaluated the consistency of the resulting delineations.

### 4.1 Individual‐Based Simulations

We simulated metacommunities using an agent‐based model formulated in continuous space and continuous time (Cornell et al. 2019). Individuals are modelled as discrete agents and their births, deaths, and movements are determined by processes that depend on local conditions such as resource availability. In contrast to patch‐based metacommunity models, this modelling framework does not make any prior delimitation between local and regional processes. Instead, a discrete structure of local communities may or may not emerge organically from the underlying processes that operate at the level of the individuals. This feature is particularly desirable for the aims of the present study, as it allows us to assess how community structure arises from individual‐level processes without prior assumptions. Moreover, it better reflects ecological realities in continuous landscapes without clear habitat boundaries. The full technical descriptions of the agent‐based models are provided in the Appendix S2.

As a first case study, we simulated consumer‐resource dynamics with partially shared resources. In this model, consumer survival and reproduction depend on limiting resources, generating intra‐ and inter‐specific competition. We simulated the dynamics of the species communities under three types of landscapes. In *continuous homogeneous landscapes*, different types of resource particles were homogeneously distributed across the landscape. In *continuous heterogeneous landscapes*, habitat patches differed in terms of the types of resources they produced. In *patchy landscapes*, the landscape consisted of discrete habitat patches, each generating all types of resources. These scenarios were selected because they correspond to the most common landscape types we encountered in the literature review. To assess the role of dispersal limitation, we also considered three movement scenarios for the consumer individuals: in the *short* scenario, all species followed short‐distance dispersal; in the *long* scenario, all species followed long‐distance dispersal. In the *mixed* scenario, half of the species followed short‐distance and the other half long‐distance dispersal.

As a second case‐study, we extended the consumer‐resource model to include host–parasite dynamics. We assumed parasites to infect consumer hosts, increasing host mortality rate relative to healthy individuals. Similarly to assuming that the hosts depend on partially shared resources, we assumed the parasites depend on partially shared host species, creating indirect effects among the host species. The second case study was specifically devised to explore whether the above‐described definitions can be applied also to asymmetric interactions and to examine to what extent the delineated local communities depend on the subset of species that are considered (e.g., hosts only or both hosts and parasites). We implemented the second case study only for the intermediate scenario of a continuous heterogeneous landscape with mixed dispersal.

### 4.2 Delineating Local Communities From Agent‐Based Simulations

In both case‐studies, we used a snapshot representing the stationarity state of the model as the current state for which we aimed to delineate the local communities. To estimate the metacommunity adjacency matrices, we ran replicate simulations starting from this fixed initial condition, of which we collected the following data. First, to follow the space‐use based definition, we discretized the simulation domain into a rectangular grid and estimated $C_{i}\left(x,y\right)$ (the space use of individual i) as the proportion of time that the individual spent in each grid cell. We then approximated $p_{\mathrm{ij}}=\int \sqrt{C_{i}\left(x,y\right)C_{j}\left(x,y\right)}\text{dxdy}$ by a discrete summation. Second, following the interaction‐based definition, we recorded all consumer‐consumer, parasite–parasite and consumer‐parasite interactions. We considered a parasite and consumer to interact if the parasite infected the host. We considered two consumers to interact if one of them consumed a resource particle that, at the time of consumption, was within the consumption radius of another individual that could have used the same resource type. Similarly, we considered two parasites to interact if one of them infected a host that the other one could have infected as well. We defined $p_{\mathrm{ij}}$ as the proportion of the replicate simulations in which an interaction took place between individuals i and j. To follow the influence‐based definition, we implemented the treatment‐control experiment illustrated in Figure 2C. Thus, for each replicate, we performed both a control simulation where the individual i was present, as well as a treatment simulation where the focal individual i was removed. In case the focal individual was an infected consumer, we also removed the parasite, as we assumed the parasites not to be free‐living. We then defined $p_{\mathrm{ij}}$ as the difference (treatment minus control) in the number of offspring produced by an individual j, averaged over the replicate simulations. In all cases, we increased the number of replicate simulations until the estimated adjacency matrix converged (see Appendix S2 for details on the convergence criteria).

We partitioned the adjacency matrices into local communities with the Leiden algorithm (Traag et al. 2019), as implemented in the R package *leidenAlg* (Kharchenko et al. 2023). The Leiden algorithm identifies the optimal partition by modularity maximisation. It can be applied to both symmetric and asymmetric adjacency matrices and to matrices where adjacencies between individuals are characterised either by presence‐absence data or by continuously varying intensities (Traag et al. 2019). We evaluated the spatial extent of the resulting local communities by calculating for different distance classes the proportion of pairs of individuals that were partitioned to the same local community. To evaluate the impact of niche overlap on the delineation of local communities, we distinguished between pairs of individuals with no resource use overlap, partial resource use overlap and complete resource use overlap.

### 4.3 Evaluating the Consistency Among Definitions to Delineate Local Communities

To evaluate the extent to which the three alternative definitions (based on space‐use, interactions and influences) result in discrete and well‐defined communities, we used the modularity score obtained from the partitioning algorithm. As a measure of consistency of the delineations obtained by two different definitions, we used a cross‐modularity index, which quantifies the agreement between different definitions' communities in terms of their internal structure. We denote by $Q\left(M_{A},P_{B}\right)$ the modularity of the adjacency matrix resulting from definition A using the partition resulting from definition B, where A and B are alternative definitions of local communities. As the Leiden algorithm seeks for a partition that maximises the modularity score, it always holds that $Q\left(M_{A},P_{B}\right)$ $\leq Q\left(M_{A},P_{A}\right)$. We define the cross‐modularity $Q_{\mathrm{AB}}$ of the adjacency matrix $M_{A}$ with respect to the partition $P_{B}$ as the proportion of its maximal modularity:

$$Q_{\mathrm{AB}}=\frac{Q\left(M_{A},P_{B}\right)\;}{Q\left(M_{A},P_{A}\right)\;}.$$

**FIGURE 4:**
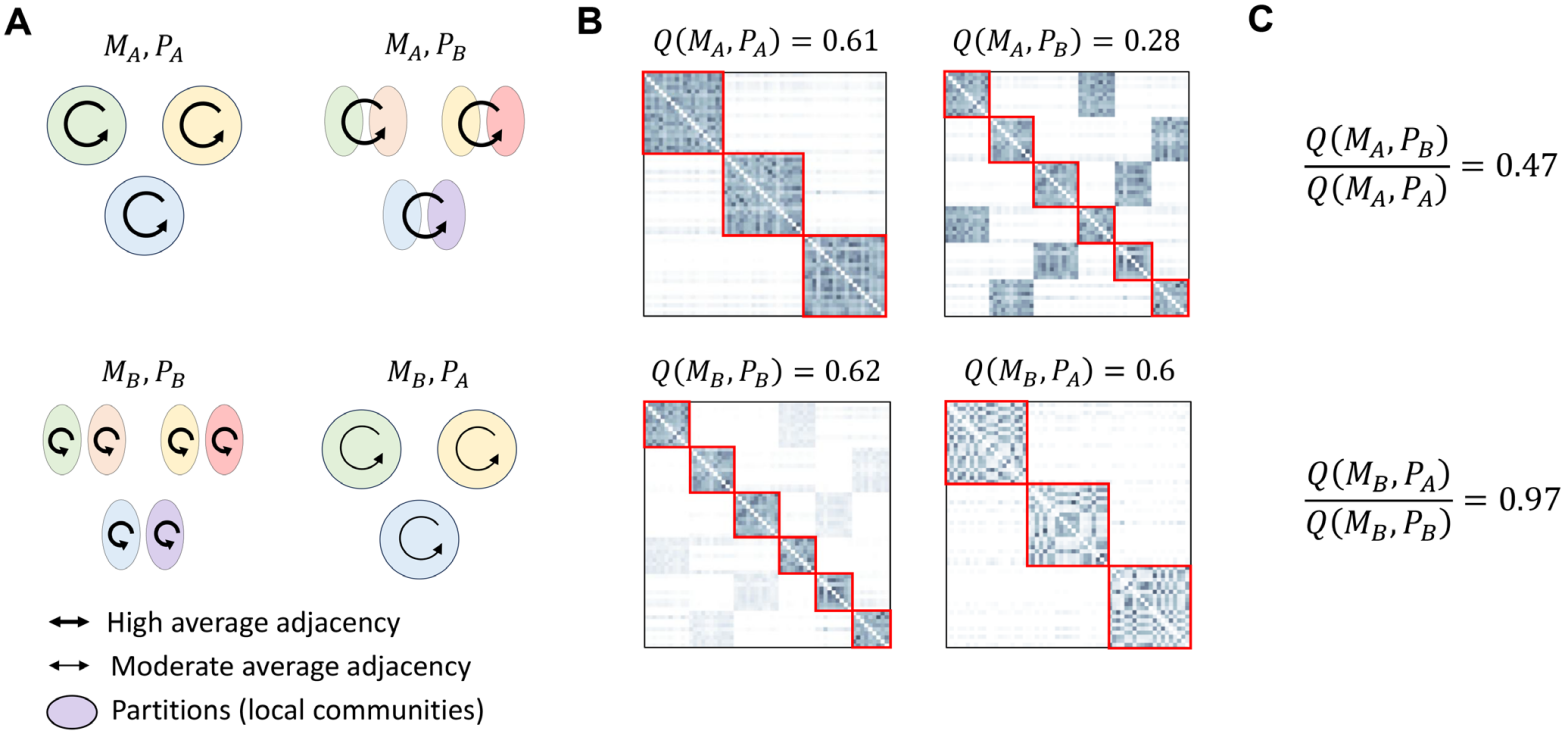
Cross‐modularity as a measure of consistency among two delineations of local communities. In this hypothetical example, the local communities delineated by definition A are larger than those delineated by definition B, which are nested within those delineated by definition A. In panel A, circles illustrate local communities and arrow widths indicate average adjacency among sets of individuals. Panel B shows the corresponding adjacency matrices ($M_{A}$ or $M_{B}$) ordered according to the delineated local communities (partition $P_{A}$ or $P_{B}$). Darker shadings indicating higher $p_{\mathrm{ij}}$ values and red squares separate the adjacency values connecting individuals partitioned to the same local community. In this example, the two adjacency matrices have nearly equal modularity, $Q\left(M_{A},P_{A}\right)\approx Q\left(M_{B},P_{B}\right)$, reflecting the similarly high adjacency within the delineated local communities and low adjacency among the delineated local communities. When the partition $P_{B}$ is used to evaluate the cross‐modularity of the adjacency matrix $M_{A}$, the value decreases by 53%. This is because many of the high adjacencies in $M_{A}$ span across the local communities of $P_{B}$. In contrast, applying partition $P_{A}$ to the adjacency matrix $M_{B}$ results in minimal decrease in modularity, because all high adjacency values are still within the diagonal blocks. Therefore, in this example, the adjacencies of A do not support the metacommunity structure of B, whereas the adjacencies from B are consistent with the broader communities of A.

Such defined cross‐modularity quantifies to what extent the communities obtained from definition B explain the variation in the adjacencies of A, relative to the local community structure that best describes A (Figure 4). Values close to 1 indicate that the two definitions lead to consistent local communities, whereas for values close to 0 this is not the case. We note that the cross‐modularity measure is not necessarily symmetric (see Figure 4 for an example where $Q_{\mathrm{AB}}\neq Q_{\mathrm{BA}}$).

### 4.4 Results for the Consumer‐Resource Model

**FIGURE 5:**
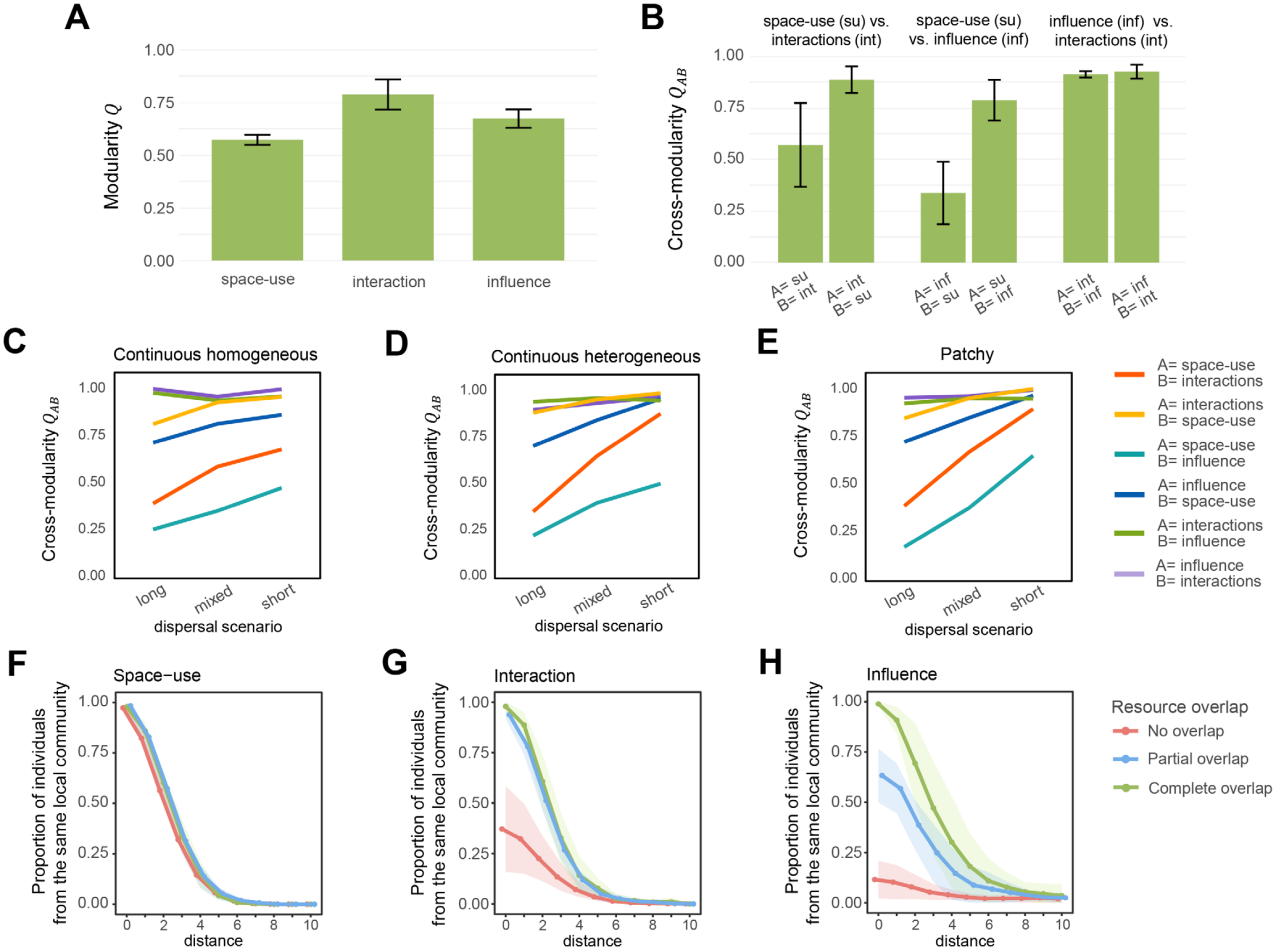
Results from the resource‐consumer model. Panels A (modularities) and B (cross‐modularities between definitions) show the mean (the green bar) ± one standard error (the black error bars) over the simulated scenarios. Panels CDE show how the cross‐modularity values depend on the simulated scenario, the panels referring to landscape types and the *x*‐axes to dispersal scenarios. Panels FGH show distance decays of proportion of individuals belonging to the same local community for different definitions of local communities: Space‐use (F), interaction (G) and influence (H). In these panels, the colours distinguish consumers by the extent of overlap in their resource use. The lines show the averages over the simulation scenarios and the shaded areas show ± one standard deviation.

In the consumer‐resource model, the interaction approach produced the highest, the influence‐based approach intermediate and the space‐use approach the lowest modularity scores (Figure 5A). Thus, accounting not only for space use but also for species interactions led the individuals to have stronger adjacencies within their local communities relative to the adjacencies between the local communities. This suggests that species interactions can refine and strengthen the local community boundaries, even if already dispersal limitation is sufficient to generate a modular organisation in spatial networks (Gilarranz 2020).

The cross‐modularity scores show that the partitions of individuals into local communities were highly consistent between the interaction‐ and influence ‐based definitions, but these were largely inconsistent with the local communities resulting from the space‐use based definition (Figure 5B). The cross‐modularity scores between space‐use based definition and the other two definitions were highly non‐symmetric and were lower when the local communities were delineated by the interaction or influence‐based definitions, but modularity was evaluated for the adjacency matrix defined by space use overlap (Figure 5B). This pattern arises because interactions and influences among individuals require some degree of overlapping space use, whereas overlapping space use alone does not necessarily result in interactions or influence. These results were robust to the choice of cross‐modularity measure (see Appendix S2).

While the modularity values showed only small differences among the simulated scenarios (small error bars in Figure 5A), the cross‐modularity values showed marked differences (large error bars in Figure 5B). The cross‐modularity values showed higher similarity between definitions in short dispersal scenarios than in long dispersal scenarios, especially in patchy landscapes (Figure 5C–E). This is because short dispersal and patchy landscape structure magnify the role of spatial proximity in determining which individuals are likely to interact and consequently influence each other. In high dispersal scenarios and in continuous landscapes, resource use differences become more important in determining individuals' spatial distributions and therefore the probability of interaction. This was reflected in the distance decay in local community membership, where for the interaction‐ and influence‐based definitions, the probability of belonging to a given local community is significantly lower for individuals with partial or no resource overlap (Figure 5F–H). A difference between the interaction‐ and influence‐based definitions was that in the latter individuals with full resource overlap were more likely to be partitioned into same communities than those with partial resource overlap (Figure 5G,H), reflecting the actual outcome of individual interactions rather than just the mere possibility of an interaction.

**FIGURE 6:**
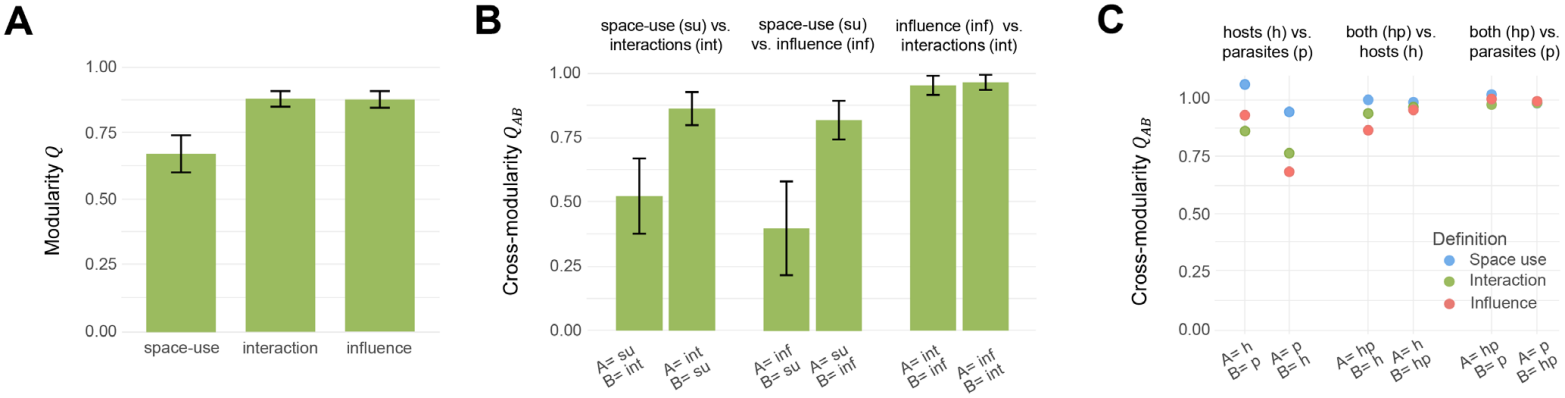
Results from the host–parasite model. Panels A (modularities) and B (cross‐modularities between definitions) show the mean (the green bar) ± one standard error (the black error bars) over the three sets of data that were used to delineate local communities: Hosts only, parasites only or both of them. Panel C show the cross‐modularities between data sets, with the colour indicating whether the applied definition of local community is based on space‐use (blue), interaction (green) or influence (red).

### 4.5 Results for the Host–Parasite Model

Let us first assume that the researchers would not be aware of the parasites and delineate the local communities based on data on the hosts only. For this case, we obtained higher modularity scores than for the baseline model without parasites: inclusion of parasites increased the modularity of the consumers from 0.54 to 0.62 for space use, from 0.80 to 0.87 for interactions and from 0.73 to 0.84 for influence. This suggests that the parasites influenced the dynamics of their hosts in a way that made local community structure more cohesive.

Let us then assume that the researchers would be aware of not only the hosts but also the parasites. They would then have three options on what data to use for delineating the local communities: hosts only, parasites only or both hosts and parasites. In all three cases, we obtained qualitatively similar results as reported above for the consumer‐resource model. Namely, the space‐use approach produced much lower modularity scores than the influence‐ and interaction ‐based approaches (Figure 6A). Furthermore, the interaction‐ and influence‐based definitions resulted in highly consistent delineations, whereas the communities delineated by the interaction‐ or influence‐based definition were essentially subsets of those obtained from the space‐use based definition (Figure 6B).

Empirical studies are typically restricted to one or few taxonomical or functional groups of species, likely representing only a subset of all those species that co‐occur in space and potentially interact. From this point of view, a relevant question to ask is how much the delineation of local communities then depends on the particular group of species. In our simulated case study, defining local communities based on shared space use resulted in consistent delineations, regardless of whether data on hosts or parasites were used (Figure 6C). However, when defining local communities based on interactions or influences, then local communities delineated based on hosts represented poorly the adjacencies of the parasites (Figure 6C), suggesting that the parasites interacted with each other not only within but also across local communities of the hosts. When delineating the local communities using data on both the hosts and the parasites, they represented well the adjacencies of both groups of species, irrespective of the definition used (Figure 6C).

## 5 Discussion

Since its formulation, Metacommunity Theory has been instrumental in understanding biodiversity dynamics (Cottenie 2005; Heino et al. 2015; Leibold and Chase 2018). A central aspect of the broad applicability of MT lies in its recognition of spatially dependent dynamic processes that link local communities across space. However, even if local communities are a central concept in MT, how to define a local community remains largely unresolved, hindering the understanding of spatial dependency of community assembly processes. Our literature review demonstrated that empirical studies framed within MT rarely explicitly define what they consider as local communities, nor do they justify their choice from an ecological point of view. In studies conducted within patchy landscapes, a typical assumption is that each habitat patch corresponds to a local community, overlooking the possibility that individuals may frequently move and interact across multiple patches or that a single patch may host multiple local communities. In studies conducted within continuous landscapes, the sampling design is often implicitly assumed to reflect local communities, with only a minority of studies explicitly justifying this choice. Our findings suggest a strong mismatch between theoretical and empirical research in metacommunity ecology, highlighting the need for a systematic approach for explicitly defining and delineating local communities in empirical studies.

There is no full consensus in metacommunity ecology or more generally in community ecology, about how local communities should be defined, for example, whether it is sufficient that populations of two or more species occupy the same geographical area at the same time or whether those species should also potentially interact with each other (Stroud et al. 2015). To understand the consequences of different ways of conceptualising local communities, we considered definitions of local communities that operationalised these two contrasting points of view. Unsurprisingly, our results demonstrate that these two viewpoints lead to different delineations of local communities, with those defined by space use only resulting in larger local communities than those also accounting for species interactions. Reassuringly, our two alternative ways of accounting for species interactions resulted in consistent delineations of local communities, suggesting that it does not make a major difference whether one accounts for the presence of interactions or the eventual influence of the interactions on the fitness of individuals.

A controversial topic when defining ecological communities is whether the communities form spatially delineable and static units (Clementsian perspective) or whether their spatial boundaries are continuous and dynamic (Gleasonian perspective). A key feature of our framework is that we did not assume any pre‐existing structure for the local communities, as is commonly done in patch‐based theoretical studies (Leibold and Chase 2018). Instead, local communities organically emerged from the individual‐level processes and we quantified to what extent they formed distinct units through measuring their modularity and the distance decay in the proportion of individuals belonging to the same local community. We showed that in some cases the boundaries of the local communities were well defined (indicated by high modularity and a steep distance decay, aligning with the Clementsian perspective), whereas in other cases, defining local communities as distinct units was not possible (indicated by low modularity and gradual distance decay, aligning with the Gleasonian perspective). The extent to which local communities formed discrete units depended on all aspects we considered: landscape structure, species dispersal, which taxonomic group was considered and which definition of local community was assumed. In our simulations we delineated local communities from a snapshot of a model's stationary state. Thus, we did not address here the question of how static or dynamic local communities are, leaving this aspect of the Clementsian versus Gleasonian perspective for future work. Based on our literature review, empirical research seldom explicitly considers the possibility that local communities may overlap in space or in their memberships.

While our agent‐based simulations clarified the conceptualisation of local communities, we acknowledge that their direct application to empirical systems is challenging, as following either the space‐use or the interaction ‐based definitions would require full tracking of the underlying processes, whereas following the influence ‐based definition would require replicated experiments. However, what can always be done—and what we urge empirical metacommunity studies to do—is to explicitly spell out how they conceptualise their local communities and what kind of justification (if any) there is for their delineation of the local communities. Beyond this obvious starting point, we next discuss how empirical studies could implement our theoretical definitions in practice.

Among our three alternative definitions, the one related to shared space use is likely the easiest to apply empirically. Tracking individual movements enables the distinction between spatial distances within which the individuals move frequently, as compared to those that represent occasional dispersal events. Such an approach has been previously implemented in population ecology for distinguishing metapopulations from other types of spatially structured populations (Albert et al. 2013; Driscoll 2007; Hanski et al. 1994) and it could be scaled up to multispecies scenarios (Costa‐Pereira et al. 2022; Schlägel et al. 2020). When direct data on individual space use is not feasible to collect, one may indirectly infer it from species‐level information on spatial scales of movement or dispersal (Calabrese and Fagan 2004). For example, dispersal kernels, combined with data on landscape permeability, may be used to estimate the potential connectivity between the individuals (Calabrese and Fagan 2004).

Our simulations demonstrate that accounting not only for shared space use but also for species interactions makes a major difference in the delineation of local communities. The simplest approach for moving from space‐use based to interaction ‐based delineation of local communities is to consider shared space use only among those species that do interact with each other, assuming that species‐level interaction data is available. The straightforward reasoning here is that, in mobile organisms, the spatial scale of movements reveals also the spatial scale at which interactions with other individuals may take place (Guzman et al. 2019; Jeltsch et al. 2013; Schlägel et al. 2020). Similarly, in sessile organisms, the amount of overlap between the areas used by different individuals (e.g., root spread or canopy area) can be used as a proxy for the intensity of interactions (Casper et al. 2003). An example of a fuller individual‐level implementation of our interaction‐based definition is provided by Dupont et al. (2014), who tracked the movement and flower visits of marked bumblebee (*Bombus* spp.) individuals and identified subnetworks within the plant‐pollinator network. Although the number of studies that empirically estimate individual‐level interaction networks is increasing (Dupont et al. 2014; Guimarães 2020; Quintero et al. 2025), these studies have not been much related to MT. This is primarily the case because observing multi‐individual, multi‐species interactions poses many logistic and methodological constraints, especially if attempted at the scale of entire natural metacommunities.

From a theoretical perspective, we consider our influence‐based definition for delineating local communities to be most satisfactory, for three reasons: (1) it does not require one to decide what is counted as an interaction; (2) it accounts not only for the presence of interactions, but also for their relative strengths; and (3) it accounts not only for direct pairwise interactions, but also for the full chain of all indirect effects. Unfortunately, this approach appears the most difficult to apply empirically, due to the difficulty in generating replicated addition/removal experiments with identical initial conditions. Similarly with the interaction‐based definition, the most pragmatic approach is to complement individual‐level estimates of shared space use with species‐level information of how the individuals are likely to influence each other. Such species‐level estimates can be derived from field experiments, laboratory experiments or non‐manipulative observational approaches or be estimated from allometric relationships, all approaches having their pros and cons (Wootton and Emmerson 2005). Examples of experiments aimed at quantifying effects of competition include addition/removal experiments in sessile organisms (Purves and Law 2002; Vogt et al. 2010), as well as tracking the spread of a controlled environmental perturbation to the surrounding individuals (Casper et al. 2003). We hope that new technologies, such as high‐resolution tracking, biologging and parentage and kinship analyses (Ellis‐Soto et al. 2025; Flanagan and Jones 2019) will enable more generally applicable approaches for directly measuring the spatial scales over which individuals influence each other's fitness.

We conclude that there is currently a strong mismatch between theoretical and empirical studies in metacommunity ecology. This is because while Metacommunity Theory is fundamentally rooted on the concept of local communities, most empirical studies leave it implicit what those local communities are and those that explicitly define local communities seldom give justification for their definitions. We argue that delineating local communities through an ecologically grounded definition is not just of academic relevance, but of major importance for advancing our understanding of the drivers that shape how biodiversity is structured, maintained and how it is likely to change in the future. As one example, colonisation–extinction dynamics at the scale of local communities are central to the persistence, turnover and diversity patterns also at broader scales (Leibold and Chase 2018). Delineating communities at too large spatial scales aggregates multiple local communities, masking local extinctions and their counteracting by colonisation, thus leading to the underestimation of colonisation–extinction rates. Conversely, delineating local communities at too small spatial scales may overestimate rescue effects, as apparent colonisation events may actually reflect internal movement within the local community. More generally, we argue that correctly identifying the scale of local communities is a central starting point for any research targeted in understanding the roles of assembly processes that operate simultaneously over multiple spatial scales. We hope that this study not only encourages empirical metacommunity ecologists to explicitly spell out how they conceptualise local communities but also inspires future methodological research aimed at providing practical tools and guidelines for objectively delineating local communities in metacommunity studies. Finally, we highlight the need for theory that extends metacommunity theory from the classical ‘one patch one local community’ scenario to other landscape‐community scenarios, including landscapes with continuous variation in habitat quality.

## Author Contributions

Lluís Serra‐Domínguez performed the review and the simulations, analysed the simulated data and wrote the first draft of the manuscript. Otso Ovaskainen provided technical support for the simulations and analysis and revised the manuscript. Nerea Abrego conceived the original idea, contributed to the review and revised the manuscript. All authors agree to the submission of the manuscript.

## Funding

This work was supported by H2020 European Research Council (856506), Department of Biological and Environmental Sciences, University of Jyväskylä, Research Council of Finland (336212, 342374, 345110, 346492).

## Conflicts of Interest

The authors declare no conflicts of interest.

## Supporting information


**Data S1:** Supporting Information.

## Data Availability

The code used to design the simulations and analyse the resulting data is available in https://doi.org/10.5281/zenodo.15496644.
