# Supplementary material for: How do Empirical Metacommunity Ecologists (not) Define Local Communities and How Could These be Better Defined?
Source: Ecol Lett. 2025 Dec 28;28(12):e70298. doi: 10.1111/ele.70298 (PMC12745672; doi:10.1111/ele.70298)
Supplement: Supplementary file 1 — Data S1: Supporting Information. [file ELE-28-0-s001.pdf]

Appendix S1. Literature review

Supporting Table S1. Data used to create Fig. 1 of the main document. The table includes the list of articles reviewed and their classification. The different columns and values represent:

- *Environment*: The type of landscape studied. Freshwater, Marine and Terrestrial.
- *Landscape conceptualization*.
  - Patchy/Cont. (Patchy/Continuous):
    (0) The landscape consists of discrete habitat patches embedded in a matrix that is unsuitable for supporting the studied communities.
    (1) The landscape is conceptualized as continuous suitable habitat, including mosaics of habitat types, gradients of habitat quality, or homogeneous landscapes.
    (2) The landscape contains habitat patches and continuous habitat (e.g.: island-mainland system for terrestrial species).
  - Patchy support:
    (0) The authors describe the landscape as patchy but provide no justification, it is unclear that the matrix is unsuitable for the studied local communities.
    (1) The patchy landscape conceptualization is supported with references or is evidently justified (e.g.: island system for terrestrial species).
    (NA) Landscape is not patchy.
- *Results specific to patchy landscapes*: The study is framed within a patchy landscape.
  - Patch – Comm. (Patch – Community conceptualization): How the authors align the habitat patches with the local communities.
    (0) Each habitat patch is considered to hold an individual local community.
    (1) Other patch – community configurations. For example, local communities are considered to inhabit multiple habitat patches, a single habitat patch contains multiple local communities.
    (2) It is unclear how the authors define the local communities within the habitat patches.
    (NA) Landscape is not patchy.
  - Support (Support provided for the Patch – Community conceptualization:
    (0) The Patch – Community conceptualization is implicitly assumed or not justified with empirical evidence
    (1) The Patch – Community conceptualization is justified with species level data (e.g.: Spatiotemporal variation in species distributions or dispersal traits).

(2) The Patch – Community conceptualization is justified using individual level data (e.g.: tracking the movement of individuals or direct observation of individual interactions).

(NA) Landscape is not patchy.

- Disc. – Overlap. (Whether the local communities are considered to be discrete or overlapping)

(0) It is implicitly assumed that the boundaries of the local communities are discrete. Each individual belongs to a single local community.

(1) The authors provide some evidence to justify that the local communities are discrete.

(2) The authors provide some evidence to consider that the local communities are overlapping (e.g.: some individuals may be part of multiple local communities)

- *Results specific to continuous landscapes: The study is framed within landscapes of continuous suitable habitat*.
  - Samp. – Comm. (Local community conceptualization within the sampling design):

(0) The smallest scale of the sampling design is considered to be a local community (e.g.: a sample or sampling site).

(1) A collection of samples is considered to be a local community.

(2) The authors do not specify how they define the local communities within the sampling design.

(NA) Landscape is not continuous.

- Support (Support provided for the Sampling design – Community conceptualization):

(0) The relationship between the spatial scale of local communities and the sampling design is not supported by empirical evidence.

(1) The authors provide species level evidence to support how local communities are embedded within the sampling design

(2) The authors provide individual level data to support how local communities are embedded within the sampling design

(NA) Landscape is not continuous

- Disc. – Overlap. (Whether the local communities are considered to be discrete or overlapping)

(1) It is implicitly assumed that the boundaries of the local communities are discrete. Each individual belongs to a single local community.

(2) The authors provide some evidence to justify that the local communities are discrete.

(3) The authors provide some evidence to consider that the local communities are overlapping (e.g.: some individuals may be part of multiple local communities).

- *Justification / assumption of the scale of the local community*
  - For every study, we recorded what the authors considered to represent the scale of the local community. For those papers for which the choice was explicitly mentioned or justified, we also recorded it.

Paper	Environment	Landscape conceptualization		Results specific to patchy landscapes			Results specific to continuous landscapes			Justification / assumption on the scale of the local community
		Patchy/Continuous	Patchy Support	Patch-Community	Support	Discont./Overlap.	Sampling-Comm.	Support	Discont./Overlap.	
Nenzen, HK; Moor, H; O'Hara, RB; Jönsson, M; Norden, J; Ottosson, E; Snäll, T. 2025. Combining observational and experimental data to estimate environmental and species drivers of fungal metacommunity dynamics. ECOLOGY	Terrestrial	0	1	0	1	0	NA	NA	NA	They explicitly say "Each log is a resource unit 'island' or independent patch in the meta-community perspective, and the scale at which fungi interact."
Fuss, T; Bistarelli, LT; Ptacnik, R; Singer, GA. 2025. Niche partitioning in a periphyton metacommunity peaks at intermediate species richness in midsized rivers. ECOLOGY	Freshwater	1	NA	NA	NA	NA	0	0	0	Sampling sites within a river network are considered to be representative of the scale of the local habitats which represent the scale of the local communities
Cai, W; Pichler, M; Biggs, J; Nicolet, P; Ewald, N; Griffiths, RA; Bush, A; Leibold, MA; Hartig, F; Yu, DW. 2025. Assembly processes inferred from eDNA surveys of a pond metacommunity are consistent with known species ecologies. ECOGRAPHY	Freshwater	0	1	0	0	0	NA	NA	NA	Ponds were assumed to represent the scale of the freshwater vertebrate local communities
Piccini, C; Segura, A; Kruk, C; Nervi, E; González-Revello, A; Bertoglio, F; Graco-Roza, C; Sotelo-Silveira, J. 2025. Evidence for strong selective forces structuring a subtropical bacterioplankton metacommunity along 800 km from freshwater reservoir to the sea. NEW ZEALAND JOURNAL OF MARINE AND FRESHWATER RESEARCH	Freshwater	1	NA	NA	NA	NA	0	0	0	Sampling sites within a river basin were assumed to represent the scale of local communities of bacterioplankton
Convertino, M. 2025. Space sculpts time: Metacommunity risk and resilience. ECOLOGICAL INDICATORS	Terrestrial	1	NA	NA	NA	NA	0	0	0	From species distribution maps, the ~100 km x 100 km scale is considered the scale of the local community
Simoes, RO; Cardoso, TS; Pereira, AN; Paschoal, F; Luque, JL. 2025. Parasite metacommunity structure of Mullus argentinae Hubbs & Marini, 1933 (Actinopterygii: Mullidae) off Rio de Janeiro, Southeastern Brazil. ANAIS DA ACADEMIA BRASILEIRA DE CIENCIAS	Marine	0	1	0	1	0	NA	NA	NA	They explicitly say "hosts can be interpreted as patches of local parasite communities"
Godsoe, W; Allen, WJ; Waller, LP; Barratt, BIP; Flanagan, SP; Marion, ZH; Tylianakis, JM; Moltchanova, E; Dickie, IA. 2025. Rapid Shifts in Relative Abundance Obscure Temporal Diversity Changes in a Metacommunity. ECOLOGY AND EVOLUTION	Terrestrial	0	1	0	0	0	NA	NA	NA	Mesocosm experiment where a pot with several plants is considered the scale of the local community for invertebrates

Horváth, Z; Haileselasie, TH; Vad, CF; Ptacnik, R; De Meester, L. 2025. Parallels and divergences in landscape genetic and metacommunity patterns in zooplankton inhabiting soda pans. OIKOS	Freshwater	0	1	0	0	0	NA	NA	NA	Soda pans (alkaline inland ponds) are assumed to represent the scale of the local community for cladoceran zooplankton communities
Luo, YH; Ma, LL; Cadotte, MW; Seibold, S; Zou, JY; Song, J; Zheng, W; Mo, ZQ; Yu, B; Li, CY; Qian, YH; Ye, LJ; Tan, SL; Liu, J; Li, DZ; Gao, LM. 2025. Ericoid mycorrhizal fungal metacommunity facilitates closely related Rhododendron species coexistence. NEW PHYTOLOGIST	Terrestrial	0	1	0	0	0	NA	NA	NA	Plant individuals are explicitly assumed to be the habitat patches for local ericoid mycorrhizal communities
de Bonilla, EPD; Vázquez-Morales, KA; Velázquez-Velázquez, E. 2025. Bird composition of a Neotropical city of Chiapas, Mexico: has a metacommunity structure?. REVISTA DE BIOLOGIA TROPICAL	Terrestrial	2	0	2	0	0	0	0	0	Sampling points across a mosaic a landscape types (with patchy and continuous habitats embedded) are considered local communities for birds
Huang, CL; Zeleny, D; Chang-Yang, CH. 2024. Integrating several analytical methods to assess strength of ecological processes behind metacommunity assembly. OIKOS	Terrestrial	1	NA	NA	NA	NA	0	0	0	Sampling plots in forests are assumed to represent the scale of the local community for vegetation
Sacramento, PA; Simoes, NR; Alves, GHZ; Amadio, S; Padial, AA; Benedito, E. 2024. Patterns of non-migratory fish metacommunity from Neotropical floodplains: Local mechanisms, regional scale and hydrological dynamics. ECOHYDROLOGY	Freshwater	2	0	0	0	0	0	0	0	Lakes are assumed to represent the scale of the local communities for fish
Vieira, MC; de Souza, CA; Barbosa, HO; Ortega, JCG; Gomes, LF; Vieira, LCG; Bini, LM. 2024. Past community data are consistent predictors of zooplankton metacommunity structure in small reservoirs. HYDROBIOLOGIA	Freshwater	2	1	0	0	0	0	0	0	Small reservoirs that are connected by the river system are considered to represent the local scale for zooplankton communities. So is kind of patchy-continuous system
Jeliazkov, A; Chase, J. 2024. When Do Traits Tell More Than Species about a Metacommunity? A Synthesis across Ecosystems and Scales. AMERICAN NATURALIST	Terrestrial, freshwater and marine	2	0	0	0	0	1	0	0	Many datatsets are included, some with data from patchy landscapes and some from continuous, and is explicitly mentioned that the definition of the local scale is very broad in this study: "metacommunity in the broad sense as assemblages of species that potentially interact, are distributed across potentially heterogeneous landscapes, and are potentially connected by spatial processes
Dupont, YL; Greve, MB; Madsen, HB; Rasmussen, C; Timóteo, S; Olesen, JM. 2024. Structure of a metacommunity of urban bees: Species diversity and spatio-temporal modularity. BASIC AND APPLIED ECOLOGY	Terrestrial	2	1	2	0	0	0	0	0	Sampling sites across a mosaic a landscape types (with patchy and continuous habitats embedded) are considered local communities for bees
Johansson, F; Yildirim, Y; Hyseni, C; Heino, J; Höglund, J; Bini, LM. 2024. Species-genetic diversity correlation in a metacommunity of urban pond invertebrates. BASIC AND APPLIED ECOLOGY	Freshwater	0	1	0	0	0	NA	NA	NA	Ponds are considered to represent the scale for the local invertebrate communities
Parry, V; Kiemel, K; Pawlak, J; Eccard, J; Tiedemann, R; Weithoff, G. 2024. Drivers of zooplankton dispersal in a pond metacommunity. HYDROBIOLOGIA	Freshwater	0	1	0	1	0	NA	NA	NA	Mesocosm experiment where local habitat patches by taking sediments from natural ponds and the mesocosms were implicitly considered to represent the scale for the local zooplankton communities
Jiménez, M; Zavala-Hurtado, JA; Vega, E; Márquez-Haro, M. 2024. Temporal beta diversity and plant metacommunity structure dynamics over a 43-year period in an inter-tropical mexican arid region. JOURNAL OF ARID ENVIRONMENTS	Terrestrial	1	NA	NA	NA	NA	0	0	0	The scale of local plots is considered the scale of the local community for perennial plant communities
Iop, S; Caldart, VM; Vélez-Martin, E; dos Santos, TG; Prado, PI; Pillar, VD; Cechin, SZ. 2024. Niche and neutral-based processes differ in importance for	Freshwater	0	1	0	0	0	NA	NA	NA	Ponds are considered to represent the scale for the local anuran communities

common and rare species in a metacommunity of anurans in subtropical grasslands. HYDROBIOLOGIA										
López-Allendes, C; Gálvez, A; Armengol, X; Alvado, B; Castillo-Escrivá , A; Mesquita-Joanes, F; Gascón, S; Ramos-Jiliberto, R; Olmo, C. 2024. Metacommunity structures of dormant and active zooplankton in two distant mediterranean regions. FRESHWATER BIOLOGY	Freshwater	0	1	0	0	0	NA	NA	NA	Ponds are considered to represent the scale for the local zooplankton communities
Laini, A; Stubbington, R; Beermann, AJ; Burgazzi, G; Datry, T; Viaroli, P; Wilkes, M; Zizka, VMA; SaccÃ², M; Leese, F. 2023. Dissecting biodiversity: assessing the taxonomic, functional and phylogenetic structure of an insect metacommunity in a river network using morphological and metabarcoding data. EUROPEAN ZOOLOGICAL JOURNAL	Freshwater	1	NA	NA	NA	NA	1	0	0	Sampling sites across a river network are considered to represent the scale of local zooplankton communities
Terry, JCD; Langdon, W; Rossberg, AG. 2023. Codistribution as an indicator of whole metacommunity response to environmental change. ECOGRAPHY	Terrestrial	1	NA	NA	NA	NA	2	0	0	They use bird and butterfly atlas data and is unclear at what scale they assume the local communities to be
Liu, Y; Lin, L; Zhang, HX; Zhang, GF; Wu, ZJ; Zhang, YP; Kang, B. 2023. Hydrological changes regulate fish metacommunity structures and 13-diversity patterns in the floodplain lake, China. ECOLOGICAL INDICATORS	Freshwater	1	NA	NA	NA	NA	1	0	0	Sampling sites within a lake are assumed to represent the scale of local fish communities
Lu, Q; Zhang, SY; Du, JQ; Liu, Q; Dong, CX; Zhao, JD; Wang, YF; Yao, M. 2023. Multi-group biodiversity distributions and drivers of metacommunity organization along a glacial-fluvial-limnic pathway on the Tibetan plateau. ENVIRONMENTAL RESEARCH	Freshwater	1	NA	NA	NA	NA	1	0	0	Sampling sites within a river network were arbitrarily considered to be representative of the scale of the local communities of several taxa
Gilbert, JD; Márquez, FJ; Guerrero, F. 2023. Assessing the Zooplankton Metacommunity (Branchiopoda and Copepoda) from Mediterranean Wetlands in Agricultural Landscapes. DIVERSITY-BASEL	Freshwater	0	1	0	0	0	NA	NA	NA	Wetlands within terrestrial habitats are considered to represent the scale of the local zooplankton communities
Piperac, MS; Simic, V; Cvijanovic, D; Medeiros, AS; Milosevic, D. 2023. The influence of spatial processes on fish community structure: using a metacommunity framework for freshwater bioassessment. ENVIRONMENTAL SCIENCE AND POLLUTION RESEARCH	Freshwater	1	NA	NA	NA	NA	0	0	0	Sampling sites within a river network were arbitrarily considered to be representative of the scale of the local fish communities
Wei, ZH; Zhou, LZ. 2023. The impact of earlier flood recession on metacommunity diversity of wintering waterbirds at shallow lakes in the middle and lower Yangtze River floodplain. AVIAN RESEARCH	Freshwater	1	NA	NA	NA	NA	1	0	0	Within a lake system, define three distinct "local metacommunities" although it is not explained why this is the case. The local communities within are implicitly defined.
Kiemel, K; Weithoff, G; Tiedemann, R. 2023. DNA metabarcoding reveals impact of local recruitment, dispersal, and hydroperiod on assembly of a zooplankton metacommunity. MOLECULAR ECOLOGY	Freshwater	0	1	0	0	0	NA	NA	NA	Kettle holes (small ponds originating from glacier depressions) are considered to represent the scale of local zooplankton communities
Cerini, F; Vignoli, L; Blust, M; Strona, G. 2023. Functional traits predict species co-occurrence patterns in a North American Odonata metacommunity. ECOSPHERE	Freshwater	0	0	0	0	0	NA	NA	NA	Sites representing different kinds of water bodies (rivers, lakes, ponds...) are considered to represent the scale of local odonata communities
Stoczynski, L; Scott, MC; Bower, L; Peoples, BK. 2023. Effects of environment and metacommunity delineation on multiple dimensions of stream fish beta diversity. FRONTIERS IN ECOLOGY AND EVOLUTION	Freshwater	1	NA	NA	NA	NA	0	0	0	Sampling sites in a river basin are considered to represent the scale of the local fish communities
Eyster, HN; Srivastava, DS; Kreitzman, M; Chan, KMA. 2022. Functional traits and metacommunity theory reveal that habitat filtering and competition maintain bird diversity in a human shared landscape. ECOGRAPHY	Terrestrial	1	NA	NA	NA	NA	1	0	0	Sampling sites within a landscape of different habitat types are considered the scale of local bird communities
Qin, CY; Ge, YF; Gao, J; Zhou, SL; Yu, J; Wang, BX; Datry, T. 2022. Ecological drivers of macroinvertebrate metacommunity assembly in a subtropical river	Freshwater	1	NA	NA	NA	NA	0	0	0	Sampling sites within a river basin were assumed to represent local communities of macroinvertebrates

basin in the Yangtze River Delta, China. SCIENCE OF THE TOTAL ENVIRONMENT										
Vass, M; Eriksson, K; Carlsson-Graner, U; Wikner, J; Andersson, A. 2022. Co-occurrences enhance our understanding of aquatic fungal metacommunity assembly and reveal potential host-parasite interactions. FEMS MICROBIOLOGY ECOLOGY	Marine	1	NA	NA	NA	NA	1	0	0	Bays within the same coastline were considered to represent the scale of the local communities of aquatic fungi
Schulte, NO; Khan, AL; Smith, EW; Zoumplis, A; Kaul, D; Allen, AE; Adams, BJ; McKnight, DM. 2022. Blowin' in the wind: Dispersal, structure, and metacommunity dynamics of aeolian diatoms in the McMurdo Sound region, Antarctica. JOURNAL OF PHYCOLOGY	Freshwater	2	1	2	0	0	1	0	0	Sampling sites from both streams and ponds were considered to represent the scale of the local communities of diatoms
Brustolin, MC; Fonseca, G; Gallucci, F. 2022. Habitat Attributes Dictate the Roles of Dispersal and Environmental Filtering on Metacommunity Assembly at Coastal Soft-Bottom Ecosystems. ESTUARIES AND COASTS	Marine	1	NA	NA	NA	NA	1	0	0	Sampling sites along a coastal line are considered to represent the scale of the local communities of nematodes
Cruz, GM; Faria, APJ; Juen, L. 2022. Patterns and metacommunity structure of aquatic insects (Trichoptera) in Amazonian streams depend on the environmental conditions. HYDROBIOLOGIA	Freshwater	1	NA	NA	NA	NA	0	0	0	Sampling sites along stream networks are considered to represent the scale of local insect communities
Costa, NA; Cardoso, TD; da Costa-Neto, SF; Alvarez, MR; Junior, AM; Gentile, R. 2022. Helminths of sigmodontine rodents in an agroforestry mosaic in the Brazilian Atlantic Forest: Patterns and processes of the metacommunity structure. INTERNATIONAL JOURNAL FOR PARASITOLOGY-PARASITES AND WILDLIFE	Terrestrial	0	1	0	1	1	NA	NA	NA	Rodents (hosts) are considered to represent the scale of local helminth communities (parasites)
Eden, R; Manica, A; Mitchell, EG. 2022. Metacommunity analyses show an increase in ecological specialisation throughout the Ediacaran period. PLOS BIOLOGY	Marine	1	NA	NA	NA	NA	0	1	0	Fosil localities are considered to represent the scale of ediacaran local communities, and this is explicitly justified: "Ediacaran communities vary in how much they are separated in time and space, from ecological to geological time scales, and their organisms have been shown to have large dispersal ranges based on reproductive mode and species occurrence over large space and time scales. Because the connectivity of these Ediacaran communities via dispersal has been established, here we define metacommunities as sets of fossil localities (communities), which are connected by the dispersal of many species"
Shukla, R; Bhat, A. 2022. Patterns and drivers of species co-occurrence networks in a tropical stream fish metacommunity. HYDROBIOLOGIA	Freshwater	1	NA	NA	NA	NA	1	0	0	Sampling sites across a river network are considered to represent the scale of local fish communities
Stearman, LW; Schaefer, JF. 2022. Long-term minnow community trait shifts and metacommunity dynamics in a geomorphically unstable river. ECOLOGY OF FRESHWATER FISH	Freshwater	1	NA	NA	NA	NA	1	0	0	Sampling sites across a river network are considered to represent the scale of local fish communities
Budnick, WR; Mruzek, JL; Larson, CA; Passy, SI. 2021. The impacts of nutrient supply and imbalance on subcontinental co-occurrence networks and metacommunity composition of stream algae. ECOGRAPHY	Freshwater	1	NA	NA	NA	NA	0	0	0	Sampling sites across river networks are considered to represent the scale of local algae communities
Firmiano, KR; Canedo-Arguelles, M; Gutierrez-Canovas, C; Macedo, DR; Linares, MS; Bonada, N; Callisto, M. 2021. Land use and local environment affect macroinvertebrate metacommunity organization in Neotropical stream networks. JOURNAL OF BIOGEOGRAPHY	Freshwater	1	NA	NA	NA	NA	1	0	0	Sampling sites across a river network are considered to represent the scale of local macroinvertebrate communities

Grisnik, M; Grinath, JB; Walker, DM. 2021. The presence of Pseudogymnoascus destructans, a fungal pathogen of bats, correlates with changes in microbial metacommunity structure. SCIENTIFIC REPORTS	Terrestrial	0	1	0	1	1	NA	NA	NA	Bat hosts are justified as the scale of local microbiome communities
Albero, L; Martinez-Solano, I; Arias, A; Lizana, M; Becares, E. 2021. Amphibian Metacommunity Responses to Agricultural Intensification in a Mediterranean Landscape. LAND	Freshwater	0	1	0	0	0	NA	NA	NA	Ponds within a terrestrial landscape are considered to represent the cale of local amphibian communities
Jiang, XM; Xu, X; Tao, M; Li, ZF; Zhang, SY; Li, HT. 2021. The effects of dispersal ability on metacommunity structure of macroinvertebrates in subtropical Chinese high-mountain streams: seasonal shifts in relative contribution of local environment and spatial processes. ENVIRONMENTAL SCIENCE AND POLLUTION RESEARCH	Freshwater	1	NA	NA	NA	NA	1	0	0	Sampling sites across a river network are considered to represent the scale of local macroinvertebrate communities
Resasco, J; Fletcher, RJ. 2021. Accounting for connectivity alters the apparent roles of spatial and environmental processes on metacommunity assembly. LANDSCAPE ECOLOGY	Terrestrial	0	0	0	0	0	NA	NA	NA	Cleared patches within a forest landscape are considered to represent the scale of local ant communities
Stoczynski, L; Brown, BL; Midway, SR; Peoples, BK. 2021. Landscape features and study design affect elements of metacommunity structure for stream fishes across the eastern USA. FRESHWATER BIOLOGY	Freshwater	1	NA	NA	NA	NA	1	0	0	Sampling sites across river networks are considered to represent the scale of local fish communities
Tan, K; Wang, CC; Li, Q; Zhang, D; Chu, L; Yan, YZ. 2021. Variation in processes structuring fish assemblages as inferred from metacommunity analyses: Differences between headwater and adventitious streams within a river network. FRESHWATER SCIENCE	Freshwater	1	NA	NA	NA	NA	0	0	0	Sampling sites across river networks are considered to represent the scale of local fish communities
Malecka-Adamowicz, M; Kubera, L. 2021. Patterns of Structural and Functional Bacterioplankton Metacommunity along a River under Anthropogenic Pressure. SUSTAINABILITY	Freshwater	1	NA	NA	NA	NA	0	0	0	Sampling sites along a river are considered to represent the scale of local bacterioplankton communities
Zheng, XF; Zhang, KK; Yang, T; He, ZL; Shu, LF; Xiao, FS; Wu, YJ; Wang, BH; Yu, H; Yan, QY. 2021. Sediment resuspension drives protist metacommunity structure and assembly in grass carp (Ctenopharyngodon idella) aquaculture ponds. SCIENCE OF THE TOTAL ENVIRONMENT	Freshwater	0	1	0	1	1	NA	NA	NA	Artificial ponds are considered to represent the scale of local protist communities
Bolnick, DI; Resetarits, EJ; Ballare, K; Stuart, YE; Stutz, WE. 2020. Host patch traits have scale-dependent effects on diversity in a stickleback parasite metacommunity. ECOGRAPHY	Freshwater	0	1	0	1	1	NA	NA	NA	Hosts (fish) are considered the local habitat patches for parasite local communities and this is explicitly mentioned: "For parasites, each individual host represents a transient habitat patch that often supports a multi-species parasite community"
He, SW; Soininen, J; Deng, GP; Wang, BX. 2020. Metacommunity Structure of Stream Insects across Three Hierarchical Spatial scales. ECOLOGY AND EVOLUTION	Freshwater	1	NA	NA	NA	NA	0	0	0	Sampling sites along river networks are considered to represent the scale of local insect communities
Wepfer, PH; Nakajima, Y; Hui, FKC; Mitarai, S; Economo, EP. 2020. Metacommunity ecology of Symbiodiniaceae hosted by the coral Galaxea fascicularis. MARINE ECOLOGY PROGRESS SERIES	Marine	0	1	0	1	0	NA	NA	NA	Coral individuals (hosts) considered as the local habitat patches for their endosymbionts
Ceron, K; Santana, DJ; Lucas, EM; Zocche, JJ; Provete, DB. 2020. Climatic variables influence the temporal dynamics of an anuran metacommunity in a nonstationary way. ECOLOGY AND EVOLUTION	Terrestrial	1	NA	NA	NA	NA	1	0	0	Sampling sites across a forest landscape considered to represent the scale of the local communities of anurans
Murray-Stoker, D; Murray-Stoker, KM. 2020. Consistent metacommunity structure despite inconsistent drivers of assembly at the continental scale. JOURNAL OF ANIMAL ECOLOGY	Freshwater	1	NA	NA	NA	NA	0	0	0	Sampling locations across river networks considered to represent the scale of local macroinvertebrate communities

Li, B; Tan, WZ; Wen, L; Zhao, XF; Peng, BF; Yang, JF; Lu, C; Wang, YY; Lei, GC. 2020. Anthropogenic habitat alternation significantly decreases alpha- and beta-diversity of benthopelagic metacommunity in a large floodplain lake. HYDROBIOLOGIA	Freshwater	1	NA	NA	NA	NA	1	0	0	Sampling sites in floodplain lakes are considered to represent the scale of local benthopelagic communities
Rodrigues, CAS; Gurgel-Lourenco, RC; Ramos, EA; Novaes, JLC; Garcez, DS; Costa, RS; Sanchez-Botero, JI. 2020. Metacommunity organization in an intermittent river in Brazil: the importance of riverine networks for regional biodiversity. AQUATIC ECOLOGY	Freshwater	1	NA	NA	NA	NA	1	0	0	Sampling sites across a river network are considered to represent the scale of local fish communities
Dalmolin, DA; Tozetti, AM; Pereira, MJR. 2020. Turnover or intraspecific trait variation: explaining functional variability in a neotropical anuran metacommunity. AQUATIC SCIENCES	Freshwater	0	1	0	0	0	NA	NA	NA	Ponds within a terrestrial landscape are considered to represent the cale of local anuran communities
Alves, AT; Petsch, DK; Barros, F. 2020. Drivers of benthic metacommunity structure along tropical estuaries. SCIENTIFIC REPORTS	Marine	1	NA	NA	NA	NA	1	0	0	Sampling sites along estuaries are considered to represent the scale of local benthic communities
Garcia-Giron, J; Heino, J; Baastrup-Spohr, L; Clayton, J; de Winton, M; Feldmann, T; Fernandez-Alaez, C; Ecke, F; Hoyer, MV; Kolada, A; Kosten, S; Lukacs, BA; Mormul, RP; Rhazi, L; Rhazi, M; Sass, L; Xu, J; Alahuhta, J. 2020. Elements of lake macrophyte metacommunity structure: Global variation and community-environment relationships. LIMNOLOGY AND OCEANOGRAPHY	Freshwater	0	1	0	0	0	NA	NA	NA	Lakes are considered to represent the scale of local macroalgae communities and give some justification for this given the spatial distances among the studied lakes
Aarnio, S; Teittinen, A; Soininen, J. 2019. High diatom species turnover in a Baltic Sea rock pool metacommunity. MARINE BIODIVERSITY	Marine	0	1	0	1	1	NA	NA	NA	Rock pools considered to represent the scale of local diatom communities. It is justified that the pools are small, not connected, and diatoms are do not mobile to use different patches
Beng, KC; Corlett, RT. 2019. Identifying the mechanisms that shape fungal community and metacommunity patterns in Yunnan, China. FUNGAL ECOLOGY	Terrestrial	1	NA	NA	NA	NA	0	0	0	Sampling sites across a forest landscape considered to represent the scale of the local communities of soil fungal communities
Guo, YX; Gao, MX; Liu, J; Zaitsev, AS; Wu, DH. 2019. Disentangling the drivers of ground-dwelling macro-arthropod metacommunity structure at two different spatial scales. SOIL BIOLOGY & BIOCHEMISTRY	Terrestrial	1	NA	NA	NA	NA	1	0	0	Sampling sites across a terrestrial landscape were considered to represent the scale of local soil invertebrate communities
Cleary, DFR; Swierts, T; Coelho, FJRC; Polonia, ARM; Huang, YSM; Ferreira, MRS; Putchakarn, S; Carvalheiro, L; van der Ent, E; Ueng, JP; Gomes, NCM; de Voogd, NJ. 2019. The sponge microbiome within the greater coral reef microbial metacommunity. NATURE COMMUNICATIONS	Marine	0	1	0	1	1	NA	NA	NA	Sponges (hosts) considered to represent the scale of local microbiome communities
Lawson, LP; Niedzwiecki, J; Petren, K. 2019. Darwin's finches: a model of landscape effects on metacommunity dynamics in the Galapagos Archipelago. ECOGRAPHY	Terrestrial	0	1	1	2	1	NA	NA	NA	The extent of the local communities of finches was estimated with genetic microsatellite data
Smith, JA; Dietl, GP. 2019. Molluscan metacommunity dynamics in the Colorado River estuary, Mexico before upstream water diversion. ANTHROPOCENE	Marine	1	NA	NA	NA	NA	1	0	0	Sampling sites along a estuary considered to represent the scale of the local molluscan communities
Szabo, B; Lengyel, E; Padisak, J; Stenger-Kovacs, C. 2019. Benthic diatom metacommunity across small freshwater lakes: driving mechanisms, -diversity and ecological uniqueness. HYDROBIOLOGIA	Freshwater	0	1	0	0	0	NA	NA	NA	Lakes are considered to represent the scale of local communities of benthic diatoms
Pujoni, DGF; Barros, CFD; Dos Santos, JBO; Maia-Barbosa, PM; Barbosa, FAR. 2019. Dispersal ability and niche breadth act synergistically to determine zooplankton but not phytoplankton metacommunity structure. JOURNAL OF PLANKTON RESEARCH	Freshwater	0	1	0	0	0	NA	NA	NA	Lakes are considered to represent the scale of local communities of zooplankton and phytoplankton communities

Lamy, T; Wang, SP; Renard, D; Lafferty, KD; Reed, DC; Miller, RJ. 2019. Species insurance trumps spatial insurance in stabilizing biomass of a marine macroalgal metacommunity. ECOLOGY	Marine	1	NA	NA	NA	NA	1	1	0	Sampling plots are considered to represent the scale of the local macroalgae communities and this is justified as "Spore dispersal estimates for macroalgae range from a few meters to several kilometers but generally average <1 km (Gaylord et al. 2002, Kinlan and Gaines 2003). This is much shorter than the maximum distance between our plots (75 km), suggesting that dispersal alone does not explain the high degree of spatial synchrony in our system."
Beck, S; Anderson, IC; Drigo, B; Powell, JR. 2019. A soil fungal metacommunity perspective reveals stronger and more localised interactions above the tree line of an alpine/subalpine ecotone. SOIL BIOLOGY & BIOCHEMISTRY	Terrestrial	1	NA	NA	NA	NA	1	0	0	Sampling points along alpine environments are considered to represent the scale of local soil fungal communities
Valente-Neto, F; Duraes, L; Siqueira, T; Roque, FO. 2018. Metacommunity detectives: Confronting models based on niche and stochastic assembly scenarios with empirical data from a tropical stream network. FRESHWATER BIOLOGY	Freshwater	0	1	0	0	0	NA	NA	NA	Riffles (shallow landform in rivers) are considered to represent the scale of local insect communities
Delciellos, AC; Borges, VNT; Prevedello, JA; Ribeiro, SE; Braga, C; Vieira, MV; Cerqueira, R. 2018. Seasonality in metacommunity structure: an empirical test in the Atlantic Forest. LANDSCAPE ECOLOGY	Terrestrial	2	0	0	0	0	0	0	0	Sites in forest patch remnants and in continuous forest considered to represent the scale of local communities of small mammals
de Mendoza, G; Kaivosoja, R; Gronroos, M; Hjort, J; Ilmonen, J; Karna, OM; Paasivirta, L; Tokola, L; Heino, J. 2018. Highly variable species distribution models in a subarctic stream metacommunity: Patterns, mechanisms and implications. FRESHWATER BIOLOGY	Freshwater	1	NA	NA	NA	NA	1	0	0	Sampling sites along a river network are considered to represent the scale of local insect communities
de Campos, R; Lansac-Toha, FM; da Conceicao, ED; Martens, K; Higuti, J. 2018. Factors affecting the metacommunity structure of periphytic ostracods (Crustacea, Ostracoda): a deconstruction approach based on biological traits. AQUATIC SCIENCES	Freshwater	1	NA	NA	NA	NA	0	0	0	Lakes connected by rivers considered to represent the scale of local communities for periphytic ostracods
Benito, X; Fritz, SC; Steinitz-Kannan, M; Velez, MI; McGlue, MM. 2018. Lake regionalization and diatom metacommunity structuring in tropical South America. ECOLOGY AND EVOLUTION	Freshwater	1	NA	NA	NA	NA	1	0	0	Lakes connected by rivers considered to represent the scale of local communities for diatoms
Tolonen, KT; Cai, YJ; Vilmi, A; Karjalainen, SM; Sutela, T; Heino, J. 2018. Environmental filtering and spatial effects on metacommunity organisation differ among littoral macroinvertebrate groups deconstructed by biological traits. AQUATIC ECOLOGY	Freshwater	1	NA	NA	NA	NA	1	0	0	Sampling sites within a lake are considered to represent the scale of local macroinvertebrate communities
Winemiller, KO; Taphom, DC; Kelso-Winemiller, LC; Lopez-Delgado, EO; Keppeler, FW; Montana, CG. 2018. Fish metacommunity structure in Cano Maraca, an important nursery habitat in the Western Llanos of Venezuela. NEOTROPICAL ICHTHYOLOGY	Freshwater	1	NA	NA	NA	NA	0	0	0	Sampling sites in a river basin considered to represent the scale of local fish communities
Rodil, IF; Lucena-Moya, P; Lastra, M. 2018. The Importance of Environmental and Spatial Factors in the Metacommunity Dynamics of Exposed Sandy Beach Benthic Invertebrates. ESTUARIES AND COASTS	Marine	0	1	0	0	0	NA	NA	NA	Beaches along a long coastal line considered to represent the scale of local communities of benthic invertebrates
Costa, LEN; Farias, RP; Santiago, ACP; Silva, IAA; Barros, ICL. 2018. Abiotic factors drives floristic variations of fern's metacommunity in an Atlantic Forest remnant. BRAZILIAN JOURNAL OF BIOLOGY	Terrestrial	1	NA	NA	NA	NA	0	0	0	Sampling plots within a forest considered to represent the scale of local fern communities

Gianuca, AT; Engelen, J; Brans, KI; Hanashiro, FTT; Vanhamel, M; van den Berg, EM; Souffreau, C; De Meester, L. 2018. Taxonomic, functional and phylogenetic metacommunity ecology of cladoceran zooplankton along urbanization gradients. ECOGRAPHY	Freshwater	0	0	0	0	0	NA	NA	NA	Ponds within a terrestrial landscape are considered to represent the cale of local cladoceran zooplankton communities
Castillo-Escriva, A; Aguilar-Alberola, JA; Mesquita-Joanes, F. 2017. Spatial and environmental effects on a rock-pool metacommunity depend on landscape setting and dispersal mode. FRESHWATER BIOLOGY	Freshwater	0	1	0	0	0	NA	NA	NA	Rock pools within a terrestrial landscape considered to represent the scale of local invertebrate communities
Heino, J; Soininen, J; Alahuhta, J; Lappalainen, J; Virtanen, R. 2017. Metacommunity ecology meets biogeography: effects of geographical region, spatial dynamics and environmental filtering on community structure in aquatic organisms. OECOLOGIA	Freshwater	1	NA	NA	NA	NA	1	0	0	Sampling sites across river networks are considered to represent the scale of local freshwater communities
Nieminen, M; van Nouhuys, S. 2017. The roles of trophic interactions, competition and landscape in determining metacommunity structure of a seed-feeding weevil and its parasitoids. ANNALES ZOOLOGICI FENNICI	Terrestrial	0	1	0	0	0	NA	NA	NA	Local communities defined as the host-parasitoid system (weevils and their parasitoids)
Brasil, LS; Vieira, TB; de Oliveira, JMB; Dias-Silva, K; Juen, L. 2017. Elements of metacommunity structure in Amazonian Zygoptera among streams under different spatial scales and environmental conditions. ECOLOGY AND EVOLUTION	Freshwater	1	NA	NA	NA	NA	0	0	0	Sampling sites along river networks are considered to represent the local communities of Zygoptera
Swan, CM; Johnson, A; Nowak, DJ. 2017. Differential organization of taxonomic and functional diversity in an urban woody plant metacommunity. APPLIED VEGETATION SCIENCE	Terrestrial	1	NA	NA	NA	NA	0	0	0	Sampling plots along urban landscapes are consider to represent the scale of the local plant communities
Zhao, K; Song, K; Pan, YD; Wang, LH; Da, LJ; Wang, QX. 2017. Metacommunity structure of zooplankton in river networks: Roles of environmental and spatial factors. ECOLOGICAL INDICATORS	Freshwater	1	NA	NA	NA	NA	1	0	0	Sampling sites along a river network are considered to represent the scale of the local zooplankton communities
Eros, T; Takacs, P; Specziar, A; Schmera, D; Saly, P. 2017. Effect of landscape context on fish metacommunity structuring in stream networks. FRESHWATER BIOLOGY	Freshwater	1	NA	NA	NA	NA	1	1	0	Sampling sites along a river network are considered to represent the scale of the local fish communities. They argument the choice of the sampling sites as "We believe that such a distance is ideal for testing metacommunity patterns in stream fish, because it is large enough that the populations cannot be considered as patchy populations, while they have the chance to interact via dispersal at ecological time scales (i.e. 100–102 years, several generations for most fish species, Matthews, 1998)."
Pentek, AL; Vad, CF; Zsuga, K; Horvath, Z. 2017. Metacommunity dynamics of amphibians in years with differing rainfall. AQUATIC ECOLOGY	Freshwater	0	1	0	0	0	NA	NA	NA	Waterbodies (lakes, ponds, temporary pools, marshes and streams) were considered to represent the scale of the local amphibian communities
Grainger, TN; Germain, RM; Jones, NT; Gilbert, B. 2017. Predators modify biogeographic constraints on species distributions in an insect metacommunity. ECOLOGY	Terrestrial	0	1	0	0	1	NA	NA	NA	Milkweed patches were considered the discrete habitat patches hosting milkweed-specialized insect local communities
Dala-Corte, RB; Becker, FG; Melo, AS. 2017. The importance of metacommunity processes for long-term turnover of riffle-dwelling fish assemblages depends on spatial position within a dendritic network. CANADIAN JOURNAL OF FISHERIES AND AQUATIC SCIENCES	Freshwater	1	NA	NA	NA	NA	0	0	0	Riffle sites along a river network are considered to represent the scale of local fish communities

Jaworski, CC; Thebaud, C; Chave, J. 2016. Dynamics and persistence in a metacommunity centred on the plant Antirrhinum majus: theoretical predictions and an empirical test. JOURNAL OF ECOLOGY	Terrestrial	0	1	0	1	1	NA	NA	NA	Plant populations are considered the habitat patches and therefore the scale of the local communities of pollinators and predators of the specific plant species. They provide justification "For the three trophic levels (plant, seed-predator, weevil), we assumed that each patch corresponded to a population " " The population border was considered to have been reached when no further plants were detected within 500 m of the last plant found. "
Dong, XY; Li, B; He, FZ; Gu, Y; Sun, MQ; Zhang, HM; Tan, L; Xiao, W; Liu, SR; Cai, QH. 2016. Flow directionality, mountain barriers and functional traits determine diatom metacommunity structuring of high mountain streams. SCIENTIFIC REPORTS	Freshwater	1	NA	NA	NA	NA	0	0	0	Sampling sites within river networks are considered to represent the scale of the local communities of diatoms
Datry, T; Melo, AS; Moya, N; Zubieta, J; De la Barra, E; Oberdorff, T. 2016. Metacommunity patterns across three Neotropical catchments with varying environmental harshness. FRESHWATER BIOLOGY	Freshwater	1	NA	NA	NA	NA	1	0	0	Sampling sites across river networks are considered to represent the scale of the local communities of invertebrate and fish communities
Fiore-Donno, AM; Weinert, J; Wubet, T; Bonkowski, M. 2016. Metacommunity analysis of amoeboid protists in grassland soils. SCIENTIFIC REPORTS	Terrestrial	1	NA	NA	NA	NA	1	0	0	Sampling points along grasslands soils are considered to represent the scale of the local communities of amoeboid protists
Tonkin, JD; Stoll, S; Jahnig, SC; Haase, P. 2016. Elements of metacommunity structure of river and riparian assemblages: Communities, taxonomic groups and deconstructed trait groups. ECOLOGICAL COMPLEXITY	Freshwater	1	NA	NA	NA	NA	1	0	0	Sampling sites along a river network were considered to represent the scale of the local invertebrate communities
Michelson, Andrew V.; Boush, Lisa E. Park; Pan, Jean J.. 2016. Discerning patterns of diversity from biogeographical distributions: testing models of metacommunity dynamics using non-marine ostracodes from San Salvador Island, Bahamas. HYDROBIOLOGIA	Freshwater	0	1	0	0	0	NA	NA	NA	Lakes are considered to represent the scale of local ostracode communities
Castillo-Escriva, A; Valls, L; Rochera, C; Camacho, A; Mesquita-Joanes, F. 2016. Spatial and environmental analysis of an ostracod metacommunity from endorheic lakes. AQUATIC SCIENCES	Freshwater	0	1	0	0	0	NA	NA	NA	Lakes are considered to represent the scale of local ostracode communities
Torres, KMM; Higgins, CL. 2016. Taxonomic and functional organization in metacommunity structure of stream-fish assemblages among and within river basins in Texas. AQUATIC ECOLOGY	Freshwater	1	NA	NA	NA	NA	0	0	0	Sampling sites across different river networks are considered to represent the scale of local fish communities
Almeida, RS; Cetra, M Almeida, Rodrigo S.; Cetra, Mauricio. 2016. Longitudinal gradient effects on the stream fish metacommunity. NATUREZA & CONSERVACAO	Freshwater	1	NA	NA	NA	NA	1	0	0	Sampling sites across a river network are considered to represent the scale of local fish communities
Dallas, TA; Kramer, AM; Zokan, M; Drake, JM. 2016. Ordination obscures the influence of environment on plankton metacommunity structure. LIMNOLOGY AND OCEANOGRAPHY LETTERS	Freshwater	0	1	0	0	0	NA	NA	NA	Small water bodies were considered to represent the scale of local zooplankton communities
Karna, OM; Gronroos, M; Antikainen, H; Hjort, J; Ilmonen, J; Paasivirta, L; Heino, J. 2015. Inferring the effects of potential dispersal routes on the metacommunity structure of stream insects: as the crow flies, as the fish swims or as the fox runs?. JOURNAL OF ANIMAL ECOLOGY	Freshwater	1	NA	NA	NA	NA	1	0	0	Sampling sites across a river network were considered to represent the scale of local insect communities
Koski, MH; Meindl, GA; Arceo-Gomez, G; Wolowski, M; LeCroy, KA; Ashman, TL. 2015. Plant-flower visitor networks in a serpentine metacommunity: assessing traits associated with keystone plant species. ARTHROPOD-PLANT INTERACTIONS	Terrestrial	0	1	0	0	0	NA	NA	NA	Serpentine seeps were considered to represent the scale of local specialized plant-flower visitor communities

Valanko, S; Heino, J; Westerbom, M; Viitasalo, M; Norkko, A. 2015. Complex metacommunity structure for benthic invertebrates in a low-diversity coastal system. ECOLOGY AND EVOLUTION	Marine	1	NA	NA	NA	NA	0	1	2	Sampling sites across the marine benthos were considered to represent the scale of local invertebrate communities. They justify their sampling scale choice by "In our study, we defined a site to be the appropriate scale for the population dynamics that underlie the mechanisms invoked to explain the patterns of EMS. Given the fact that species may vary in the size of their individual "local" populations, it is likely that local and regional scales may merge for different species at different spatial extents. We, however, believe that the patterns discovered in this study represent real biological gradients across the 1700 km2 study area, and do not represent error in estimating the scale of a community."
Yeh, YC; Peres-Neto, PR; Huang, SW; Lai, YC; Tu, CY; Shiah, FK; Gong, GC; Hsieh, CH. 2015. Determinism of bacterial metacommunity dynamics in the southern East China Sea varies depending on hydrography. ECOGRAPHY	Marine	0	0	0	0	0	NA	NA	NA	Sampling sites across the sea are considered to represent the scale of local bacterial communities. The samples consist of water samples so is a continuous environment, by the authors refer to "habitat patches"
Hoch, JM; Sokol, ER; Parker, AD; Trexler, JC. 2015. Migration Strategies Vary in Space, Time, and Among Species in the Small-fish Metacommunity of the Everglades. COPEIA	Freshwater	1	NA	NA	NA	NA	1	0	0	Sampling sites across a river network are considered to represent the scale of local fish communities
Valdivia, N; Aguilera, MA; Navarrete, SA; Broitman, BR. 2015. Disentangling the effects of propagule supply and environmental filtering on the spatial structure of a rocky shore metacommunity. MARINE ECOLOGY PROGRESS SERIES	Marine	1	NA	NA	NA	NA	1	0	0	Sampling plots in shore habitats along a coastal line were considered to represent the scale of local invertebrate communities
Sokol, ER; Brown, BL; Carey, CC; Tornwall, BM; Swan, CM; Barrett, JE. 2015. Linking management to biodiversity in built ponds using metacommunity simulations. ECOLOGICAL MODELLING	Freshwater	0	1	0	0	0	NA	NA	NA	Ponds were considered to represent the scale of local zooplankton communities
Cisneros, LM; Fagan, ME; Willig, MR. 2015. Season-specific and guild-specific effects of anthropogenic landscape modification on metacommunity structure of tropical bats. JOURNAL OF ANIMAL ECOLOGY	Terrestrial	1	NA	NA	NA	NA	0	0	0	Sampling sites across a mosaic of different habitat types are considered to represent the scale of local bat communities
Vuono, DC; Benecke, J; Henkel, J; Navidi, WC; Cath, TY; Munakata-Marr, J; Spear, JR; Drewes, JE. 2015. Disturbance and temporal partitioning of the activated sludge metacommunity. ISME JOURNAL	Freshwater	1	NA	NA	NA	NA	0	0	0	Water samples were considered the scale of local microbial communities
Soares, CEA; Velho, LFM; Lansac-Toha, FA; Bonecker, CC; Landeiro, VL; Bini, LM. 2015. The likely effects of river impoundment on beta-diversity of a floodplain zooplankton metacommunity. NATUREZA & CONSERVACAO	Freshwater	1	NA	NA	NA	NA	1	0	0	Sampling sites along a river were considered representative of local zooplankton communities, but is unclear
Pellowe-Wagstaff, KE; Simonis, JL. 2014. The ecology and mechanisms of overflow-mediated dispersal in a rock-pool metacommunity. FRESHWATER BIOLOGY	Freshwater	0	1	0	0	0	NA	NA	NA	Rockpools are considered to represent the scale of local phytoplankton, zooplankton and insect communities, and is explicitly mentioned as "Each represents a single habitat patch containing a local food web".
Fernandes, IM; Henriques-Silva, R; Penha, J; Zuanon, J; Peres-Neto, PR. 2014. Spatiotemporal dynamics in a seasonal metacommunity structure is predictable: the case of floodplain-fish communities. ECOGRAPHY	Freshwater	2	1	0	1	0	0	1	0	Sampling plots representing ponds or flooded areas are considered to represent the scale of the local fish communities. This is explicitly justified: "and the distance among plots is large enough for them to be

										considered as separated fish communities, as the species in the region are mostly small and do not move across sampled plots for activities such as daily foraging."
Davis, B; Baker, R; Sheaves, M. 2014. Seascape and metacommunity processes regulate fish assemblage structure in coastal wetlands. MARINE ECOLOGY PROGRESS SERIES	Marine	0	1	0	0	0	NA	NA	NA	Wetland habitat patches are considered to represent the scale of local fish communities
Valverde, A; Makhalanyane, TP; Cowan, DA. 2014. Contrasting assembly processes in a bacterial metacommunity along a desiccation gradient. FRONTIERS IN MICROBIOLOGY	Terrestrial	1	NA	NA	NA	NA	1	0	0	Samples along the soil environment were considered to represent the scale of local bacterial communities
Provete, DB; Goncalves-Souza, T; Garey, MV; Martins, IA; Rossa-Feres, DD. 2014. Broad-scale spatial patterns of canopy cover and pond morphology affect the structure of a Neotropical amphibian metacommunity. HYDROBIOLOGIA	Freshwater	0	1	0	0	0	NA	NA	NA	Ponds within a terrestrial environment are considered to represent the scale of local amphibian communities
Goncalves-Souza, T; Romero, GQ; Cottenie, K. 2014. Metacommunity versus Biogeography: A Case Study of Two Groups of Neotropical Vegetation-Dwelling Arthropods. PLOS ONE	Terrestrial	0	1	0	0	0	NA	NA	NA	Vegetation patches were considered to represent the scale of local arthropod communities
Borthagaray, AI; Arim, M; Marquet, PA. 2014. Inferring species roles in metacommunity structure from species co-occurrence networks. PROCEEDINGS OF THE ROYAL SOCIETY B-BIOLOGICAL SCIENCES	Terrestrial	0	1	0	0	0	NA	NA	NA	Vegetation patches were considered to represent the scale of local arthropod communities, and this is explicitly mentioned: "We sampled animal species in 31 local communities (i.e. patches)"
Ray, C; Collinge, SK. 2014. Quantifying the dominance of local control and the sources of regional control in the assembly of a metacommunity. ECOLOGY	Freshwater	0	1	0	0	0	NA	NA	NA	Vernal pools were considered to represent the scale of local plant communities
de la Sancha, NU; Higgins, CL; Presley, SJ; Strauss, RE. 2014. Metacommunity structure in a highly fragmented forest: has deforestation in the Atlantic Forest altered historic biogeographic patterns?. DIVERSITY AND DISTRIBUTIONS	Terrestrial	0	1	0	0	0	NA	NA	NA	Forest fragment remnants were considered to represent the scale of local small mammal communities
Simonis, JL; Ellis, JC Simonis, Joseph L.; Ellis, Julie C.. 2014. Bathing birds bias beta-diversity: Frequent dispersal by gulls homogenizes fauna in a rock-pool metacommunity. ECOLOGY	Freshwater	0	1	0	0	0	NA	NA	NA	Rock pools were considered to represent the scale of invertebrate local communities
Gothe, Emma; Angeler, David G.; Gottschalk, Steffi; Lofgren, Stefan; Sandin, Leonard. 2013. The Influence of Environmental, Biotic and Spatial Factors on Diatom Metacommunity Structure in Swedish Headwater Streams. PLOS ONE	Freshwater	1	NA	NA	NA	NA	1	0	0	Sampling sites along river networks were considered to represent the scale of local diatom communities
Gronroos, M; Heino, J; Siqueira, T; Landeiro, VL; Kotanen, J; Bini, LM. 2013. Metacommunity structuring in stream networks: roles of dispersal mode, distance type, and regional environmental context. ECOLOGY AND EVOLUTION	Freshwater	1	NA	NA	NA	NA	1	0	0	Sampling sites along river networks were considered to represent the scale of local macroinvertebrate communities
Funk, A; Schiemer, F; Reckendorfer, W. 2013. Metacommunity structure of aquatic gastropods in a river floodplain: the role of niche breadth and drift propensity. FRESHWATER BIOLOGY	Freshwater	1	NA	NA	NA	NA	1	0	0	A river section (pooled sampling data) was considered the scale that represents the scale of local gastropod communities
Alahuhta, J; Heino, J. 2013. Spatial extent, regional specificity and metacommunity structuring in lake macrophytes. JOURNAL OF BIOGEOGRAPHY	Freshwater	0	1	2	0	0	NA	NA	NA	Lakes were considered to represent the scale that represents local macrophyte communities
Baiser, B; Buckley, HL; Gotelli, NJ; Ellison, AM. 2013. Predicting food-web structure with metacommunity models. OIKOS	Terrestrial	0	1	0	1	1	NA	NA	NA	A carnivorous plant was considered the "patch". Interacting species are aquatic larvae, bacteria and

										protozoa. It is justified that the "patches" are small and the species are not mobile to use different "patches".
Halme, P; Odor, P; Christensen, M; Piltaver, A; Veerkamp, M; Walleyn, R; Siller, I; Heilmann-Clausen, J. 2013. The effects of habitat degradation on metacommunity structure of wood-inhabiting fungi in European beech forests. BIOLOGICAL CONSERVATION	Terrestrial	0	1	0	0	0	NA	NA	NA	Dead logs were considered to represent the scale of local wood-inhabiting fungal communities
Gothe, E; Angeler, DG; Sandin, L. 2013. Metacommunity structure in a small boreal stream network. JOURNAL OF ANIMAL ECOLOGY	Freshwater	1	NA	NA	NA	NA	1	0	0	Sampling sites within a river network were considered to represent the scale of local benthic invertebrate communities
Simonis, JL. 2013. Prey (Moina macrocopa) population density drives emigration rate of its predator (Trichocorixa verticalis) in a rock-pool metacommunity. HYDROBIOLOGIA	Marine	0	1	0	0	0	NA	NA	NA	Rock pools were considered to represent the scale of local communities of insect predator-prey systems
Richgels, Katherine L. D.; Hoverman, Jason T.; Johnson, Pieter T. J.. 2013. Evaluating the role of regional and local processes in structuring a larval trematode metacommunity of Helisoma trivolvis. ECOGRAPHY	Freshwater	0	1	0	1	0	NA	NA	NA	Hosts are considered to represent the scale of local internal parasite communities
Meynard, CN; Lavergne, S; Boulangeat, I; Garraud, L; Van Es, J; Mouquet, N; Thuiller, W. 2013. Disentangling the drivers of metacommunity structure across spatial scales. JOURNAL OF BIOGEOGRAPHY	Terrestrial	1	NA	NA	NA	NA	0	0	0	Sampling plots are considered to represent the scale of local plant communities
Brunbjerg, AK; Ejrnaes, R; Svenning, JC. 2012. Species sorting dominates plant metacommunity structure in coastal dunes. ACTA OECOLOGICA-INTERNATIONAL JOURNAL OF ECOLOGY	Terrestrial	1	NA	NA	NA	NA	1	0	0	Sampling sites in dunes along the coastal line were considered to represent the scale of local plant communities
Newton, Adrian C.; Walls, Robin M.; Golicher, Duncan; Keith, Sally A.; Diaz, Anita; Bullock, James M.. 2012. Structure, composition and dynamics of a calcareous grassland metacommunity over a 70-year interval. JOURNAL OF ECOLOGY	Terrestrial	1	NA	NA	NA	NA	0	0	0	Sampling sites across grasslands were considered to represent the scale of local plant communities
Schulz, G; Siqueira, T; Stefan, G; Roque, FD. 2012. Passive and active dispersers respond similarly to environmental and spatial processes: an example from metacommunity dynamics of tree hole invertebrates. FUNDAMENTAL AND APPLIED LIMNOLOGY	Freshwater	0	1	0	1	1	NA	NA	NA	Water filled tree holes are considered to represent the scale for local invertebrate communities, and they explicitly justify it: "their relatively small size and clearly defined boundaries, which allow investigators to determine where the habitat begins and ends, solving one of the problems associated with the study of local communities"
Eros, T; Saly, P; Takacs, P; Specziar, A; Biro, P. 2012. Temporal variability in the spatial and environmental determinants of functional metacommunity organization - stream fish in a human-modified landscape. FRESHWATER BIOLOGY	Freshwater	1	NA	NA	NA	NA	1	0	0	Sampling sites along a river system are considered to represent the scale of local fish communities
Lopez-Gonzalez, C; Presley, SJ; Lozano, A; Stevens, RD; Higgins, CL. 2012. Metacommunity analysis of Mexican bats: environmentally mediated structure in an area of high geographic and environmental complexity. JOURNAL OF BIOGEOGRAPHY	Terrestrial	1	NA	NA	NA	NA	1	0	0	Sampling sites across a terrestrial landscape are considered to represent the scale of local bat communities
Dejenie, T; Declerck, SAJ; Asmelash, T; Risch, S; Mergeay, J; De Bie, T; De Meester, L. 2012. Cladoceran community composition in tropical semi-arid highland reservoirs in Tigray (Northern Ethiopia): A metacommunity perspective applied to young reservoirs. LIMNOLOGICA	Freshwater	0	1	0	0	0	NA	NA	NA	Water reservoirs are considered to represent the scale of local cladoceran communities

Langenheder, Silke; Berga, Merce; Ostman, Orjan; Szekely, Anna J.. 2012. Temporal variation of beta-diversity and assembly mechanisms in a bacterial metacommunity. ISME JOURNAL	Marine	0	1	0	0	0	NA	NA	NA	Rockpools were considered to represent the scale of local bacterial communities
Sebastian-Gonzalez, Esther; Molina, Jose A.; Paracuellos, Mariano. 2012. Distribution patterns of a marsh vegetation metacommunity in relation to habitat configuration. AQUATIC BIOLOGY	Freshwater	0	1	0	0	0	NA	NA	NA	Marshes were considered to represent the scale of local specialized plant communities
De Bie, T; De Meester, L; Brendonck, L; Martens, K; Goddeeris, B; Ercken, D; Hampel, H; Denys, L; Vanhecke, L; Van der Gucht, K; Van Wichelen, J; Vyverman, W; Declerck, SAJ. 2012. Body size and dispersal mode as key traits determining metacommunity structure of aquatic organisms. ECOLOGY LETTERS	Freshwater	0	1	0	0	0	NA	NA	NA	Farmland ponds were considered to represent the scale of the local communities of multiple taxa: bacteria, phytoplankton, phytobenthos, cladocerans, rotifers, macrophytes, molluscs, chironomids, heteropterans, coleopterans, amphibians and fish
Frisch, D; Cottenie, K; Badosa, A; Green, AJ. 2012. Strong Spatial Influence on Colonization Rates in a Pioneer Zooplankton Metacommunity. PLOS ONE	Freshwater	0	1	0	0	0	NA	NA	NA	Ponds were considered to represent the scale of the local zooplankton communities
Chisholm, C; Lindo, Z; Gonzalez, A. 2011. Metacommunity diversity depends on connectivity and patch arrangement in heterogeneous habitat networks. ECOGRAPHY	Terrestrial	2	1	0	1	0	0	1	0	Experimental moss habitat patches are considered to delimit the size of local microarthropod communities
Sanvicente-Añorve, L; Sánchez-Ramírez, M; Ocaña-Luna, A; Flores-Coto, C; Ordoñez-López, U. 2011. Metacommunity structure of estuarine fish larvae: the role of regional and local processes. JOURNAL OF PLANKTON RESEARCH	Marine	1	NA	NA	NA	NA	1	0	0	Sampling sites in lagoons were considered to represent the scale of the local communities of fish larvae
Patrick, Christopher J.; Swan, Christopher M.. 2011. Reconstructing the assembly of a stream-insect metacommunity. JOURNAL OF THE NORTH AMERICAN BENTHOLOGICAL SOCIETY	Freshwater	1	NA	NA	NA	NA	1	0	0	Sampling sites in river networks were considered to represent the local scale of invertebrates
Welsh, HH; Hodgson, GR. 2011. Spatial relationships in a dendritic network: the herpetofaunal metacommunity of the Mattole River catchment of northwest California. ECOGRAPHY	Freshwater	1	NA	NA	NA	NA	0	0	0	River sections were considered to represent the local scale of herpetofauna
Convertino, M. 2011. Neutral metacommunity clustering and SAR: River basin vs. 2-D landscape biodiversity patterns. ECOLOGICAL MODELLING	Freshwater	1	NA	NA	NA	NA	2	0	0	Sampling sites/plots along river networks are considered to represent the scale of local tree and fish communities
Hovatter, Stephanie R.; Dejelo, Chris; Case, Andrea L.; Blackwood, Christopher B.. 2011. Metacommunity organization of soil microorganisms depends on habitat defined by presence of Lobelia siphilitica plants. ECOLOGY	Terrestrial	1	NA	NA	NA	NA	1	0	0	Soil samples were considered to represent the scale of local microbial communities
Aström, J; Bengtsson, J. 2011. Patch size matters more than dispersal distance in a mainland-island metacommunity. OECOLOGIA	Terrestrial	0	1	0	0	0	NA	NA	NA	Experimental moss patches in bedrock were considered to represent the scale of local mite and springtale communities
Keith, SA; Newton, AC; Morecroft, MD; Golicher, DJ; Bullock, JM. 2011. Plant metacommunity structure remains unchanged during biodiversity loss in English woodlands. OIKOS	Terrestrial	0	1	0	0	0	NA	NA	NA	Woodland patches surrounded by farmland and urban areas are considered to represent the scale of local plant communities
Allen, MR; VanDyke, JN; Cáceres, CE. 2011. Metacommunity assembly and sorting in newly formed lake communities. ECOLOGY	Freshwater	0	1	0	0	0	NA	NA	NA	Lakes are considered the scale to represent local zooplankton communities
Presley, Steven J.; Willig, Michael R.; Bloch, Christopher P.; Castro-Arellano, Ivan; Higgins, Christopher L.; Klingbeil, Brian T.. 2011. A Complex Metacommunity Structure for Gastropods Along an Elevational Gradient. BIOTROPICA	Terrestrial	1	NA	NA	NA	NA	1	0	0	Sampling sites in woodland were considered to represent the scale of the local gastropod communities
Dorazio, RM; KÃ©ry, M; Royle, JA; Plattner, M. 2010. Models for inference in dynamic metacommunity systems. ECOLOGY	Terrestrial	1	NA	NA	NA	NA	0	0	0	Sampling quadrats from butterfly monitoring surveys are considered to represent the scale of the local communities

de Macedo-Soares, PHM; Petry, AC; Farjalla, VF; Caramaschi, EP. 2010. Hydrological connectivity in coastal inland systems: lessons from a Neotropical fish metacommunity. ECOLOGY OF FRESHWATER FISH	Marine	2	1	0	0	0	0	0	0	Sampling sites in a lagoon are considered to represent the scale of the local communities of fish
Brown, BL; Swan, CM. 2010. Dendritic network structure constrains metacommunity properties in riverine ecosystems. JOURNAL OF ANIMAL ECOLOGY	Freshwater	1	NA	NA	NA	NA	1	0	0	Sampling sites along a river network are considered to represent the scale of macroinvertebrate local communities
Caro, AU; Navarrete, SA; Castilla, JC. 2010. Ecological convergence in a rocky intertidal shore metacommunity despite high spatial variability in recruitment regimes. PROCEEDINGS OF THE NATIONAL ACADEMY OF SCIENCES OF THE UNITED STATES OF AMERICA	Marine	1	NA	NA	NA	NA	1	0	0	Sampling sites along the coast are considered to represent the scale of the macroinvertebrates local communities
He, TH; Lamont, BB. 2010. Species versus genotypic diversity of a nitrogen-fixing plant functional group in a metacommunity. POPULATION ECOLOGY	Terrestrial	1	NA	NA	NA	NA	0	0	0	Sampling plots in sand dunes were considered to represent the scale of local plant communities (of 11 focal species)
Okuda, T; Noda, T; Yamamoto, T; Hori, M; Nakaoka, M. 2010. Contribution of environmental and spatial processes to rocky intertidal metacommunity structure. ACTA OECOLOGICA-INTERNATIONAL JOURNAL OF ECOLOGY	Marine	1	NA	NA	NA	NA	1	0	0	Sampling sites along the seashore were considered to represent the scale of the local communities of multiple taxa: macroalgae, sessile invertebrates, and mobile mollusks. References are provided to support their delineation of local communities
Bergerot, B; Julliard, R; Baguette, M. 2010. Metacommunity Dynamics: Decline of Functional Relationship along a Habitat Fragmentation Gradient. PLOS ONE	Terrestrial	0	1	0	0	0	NA	NA	NA	Fragmented forest remnants surrounded by urban areas are considered to represent the scale of local communities of host-parasitoid systems
Pillar, VD; Duarte, LDS. 2010. A framework for metacommunity analysis of phylogenetic structure. ECOLOGY LETTERS	Terrestrial	1	NA	NA	NA	NA	1	0	0	Plots in grasslands are considered to represent the scale of local plant communities
Tomasovych, A; Kidwell, SM. 2010. The Effects of Temporal Resolution on Species Turnover and on Testing Metacommunity Models. AMERICAN NATURALIST	Marine	1	NA	NA	NA	NA	1	0	0	Samples of sediments or fossil records are considered to represent the scale of the local communities of ostracods and mollusks, respectively.
Leibold, Mathew A.; Economo, Evan P.; Peres-Neto, Pedro. 2010. Metacommunity phylogenetics: separating the roles of environmental filters and historical biogeography. ECOLOGY LETTERS	Freshwater	0	1	0	0	0	NA	NA	NA	Lakes are considered to represent the scale of plankton local communities
Löbel, S; Snäll, T; Rydin, H. 2009. Mating system, reproduction mode and diaspore size affect metacommunity diversity. JOURNAL OF ECOLOGY	Terrestrial	0	1	0	0	0	NA	NA	NA	Forest patches surrounded by non-forest habitats are considered to represent the scale of local forest-specialized bryophyte communities
MacNeil, MA; Graham, NAJ; Polunin, NVC; Kulbicki, M; Galzin, R; Harmelin-Vivien, M; Rushton, SP. 2009. Hierarchical drivers of reef-fish metacommunity structure. ECOLOGY	Marine	0	1	0	0	0	NA	NA	NA	Reef atolls are considered to represent the scale of local fish communities
Pandit, SN; Kolasa, J; Cottenie, K. 2009. Contrasts between habitat generalists and specialists: an empirical extension to the basic metacommunity framework. ECOLOGY	Freshwater	0	1	0	0	0	NA	NA	NA	Rock pools are considered to represent the scale of local invertebrate communities
Vanschoenwinkel, B; Gielen, S; Seaman, M; Brendonck, L. 2009. Wind mediated dispersal of freshwater invertebrates in a rock pool metacommunity: differences in dispersal capacities and modes. HYDROBIOLOGIA	Freshwater	0	1	2	0	0	NA	NA	NA	Rock pools are considered to represent the scale of local invertebrate communities
Debout, GDG; Dalecky, A; Ngomi, A; Mckey, DB . 2009. Dynamics of species coexistence: maintenance of a plant-ant competitive metacommunity. OIKOS	Terrestrial	0	1	2	0	0	NA	NA	NA	Habitat patches of a host plant are considered to represent the scale of the local communities of their associated ants

Driscoll, DA; Lindenmayer, DB. 2009. Empirical tests of metacommunity theory using an isolation gradient. ECOLOGICAL MONOGRAPHS	Terrestrial	0	0	0	0	0	NA	NA	NA	Focal habitat patches embedded within a landscape mosaic of different habitat types are considered to represent the scale of the local bird and reptile communities. It is sometimes unclear whether the habitat patches are discrete or continuous units
Presley, SJ; Higgins, CL; López-González, C; Stevens, RD. 2009. Elements of metacommunity structure of Paraguayan bats: multiple gradients require analysis of multiple ordination axes. OECOLOGIA	Terrestrial	1	NA	NA	NA	NA	0	0	0	Sampling sites across a landscape mosaic with different habitat types are considered to represent the scale of local bat communities
Burns, KC; Neufeld, CJ. 2009. Plant extinction dynamics in an insular metacommunity. OIKOS	Terrestrial	0	1	0	0	0	NA	NA	NA	Inland islands are considered to represent the local communities for woody plants
Altermatt, F; Pajunen, VI; Ebert, D. 2009. Desiccation of Rock Pool Habitats and Its Influence on Population Persistence in a Daphnia Metacommunity. PLOS ONE	Freshwater	0	1	0	0	0	NA	NA	NA	Rockpools are considered to represent the scale of daphnia local communities
Brooks, DR; Perry, JN; Clark, SJ; Heard, MS; Firbank, LG; Holdgate, R; Mason, NS; Shortall, CR; Skellern, MP; Woiwod, IP. 2008. National-scale metacommunity dynamics of carabid beetles in UK farmland. JOURNAL OF ANIMAL ECOLOGY	Terrestrial	1	NA	NA	NA	NA	1	0	0	Sampling sites in fields were considered to represent the scale of local carabid beetles
Altermatt, F; Pajunen, VI; Ebert, D. 2008. Climate change affects colonization dynamics in a metacommunity of three Daphnia species. GLOBAL CHANGE BIOLOGY	Freshwater	0	1	0	0	0	NA	NA	NA	Rock pools were considered to represent the scale of local daphnia communities
Escarguel, G; Legendre, S; Sigé, B. 2008. Unearthing deep-time biodiversity changes: The Palaeogene mammalian metacommunity of the Quercy and Limagne area (Massif Central, France). COMPTES RENDUS GEOSCIENCE	Terrestrial	1	NA	NA	NA	NA	1	0	0	Sampling localities are considered to represent the scale of the local fauna communities screened from fossils
Azeria, ET; Kolasa, J. 2008. Nestedness, niche metrics and temporal dynamics of a metacommunity in a dynamic natural model system. OIKOS	Marine	0	1	0	1	1	NA	NA	NA	Rock pools were considered to represent the scale of aquatic invertebrate communities. Given the small volume and isolation of patches, we consider justified that patches = local communities
Vanschoenwinkel, B; Gielen, S; Vandewaerde, H; Seaman, M; Brendonck, L. 2008. Relative importance of different dispersal vectors for small aquatic invertebrates in a rock pool metacommunity. ECOGRAPHY	Terrestrial	0	1	0	0	0	NA	NA	NA	Rock pools were considered to represent the scale of local invertebrate communities
Boudell, JA; Stromberg, JC. 2008. Flood pulsing and metacommunity dynamics in a desert riparian ecosystem. JOURNAL OF VEGETATION SCIENCE	Terrestrial	1	NA	NA	NA	NA	1	0	0	Sampling sites along a riparian network are considered to represent the scale of the local plant communities
Barone, JA; Thomlinson, J; Cordero, PA; Zimmerman, JK. 2008. Metacommunity Structure of Tropical Forest along an Elevation Gradient in Puerto Rico. JOURNAL OF TROPICAL ECOLOGY	Freshwater	1	NA	NA	NA	NA	0	0	0	Sampling plots along a river system are considered to represent the scale of local riparian plant communities
McCauley, SJ; Davis, CJ; Relyea, RA; Yurewicz, KL; Skelly, DK; Werner, EE. 2008. Metacommunity patterns in larval odonates. OECOLOGIA	Freshwater	0	1	0	0	0	NA	NA	NA	Lakes and ponds are considered to represent the scale of the local communities of odonates
Muneepeerakul, R; Bertuzzo, E; Lynch, HJ; Fagan, WF; Rinaldo, A; Rodriguez-Iturbe, I. 2008. Neutral metacommunity models predict fish diversity patterns in Mississippi-Missouri basin. NATURE	Freshwater	1	NA	NA	NA	NA	0	0	0	Sampling sites along a river system are considered to represent the scale of local fish communities
Munguia, P; Miller, TE. 2008. Habitat destruction and metacommunity size in pen shell communities. JOURNAL OF ANIMAL ECOLOGY	Marine	0	1	0	0	0	NA	NA	NA	Shells are assumed to represent the scale of local communities of motile species (crustaceans, fishes, barnacles and bryozoans)
Questad, EJ; Foster, BL. 2007. Vole disturbances and plant diversity in a grassland metacommunity. OECOLOGIA	Terrestrial	1	NA	NA	NA	NA	0	0	0	Sampling plots in grasslands are considered to represent the scale of the local communities of plants

Hugueny, B; Cornell, HV; Harrison, S. 2007. Metacommunity models predict the local-regional species richness relationship in a natural system. ECOLOGY	Freshwater	0	1	0	0	0	NA	NA	NA	Rock pools are considered to represent the scale of the local daphnia communities
Vanschoenwinkel, B; De Vries, C; Seaman, M; Brendonck, L. 2007. The role of metacommunity processes in shaping invertebrate rock pool communities along a dispersal gradient. OIKOS	Freshwater	0	1	0	0	0	NA	NA	NA	Rock pools are considered to represent the scale of local zooplankton communities
Forster, MA; Warton, DI. 2007. A metacommunity-scale comparison of species-abundance distribution models for plant communities of eastern Australia. ECOGRAPHY	Terrestrial	1	NA	NA	NA	NA	0	0	0	Sampling sites along a landscape mosaic of different habitat types is considered to represent the scale of local plant communities
Burns, KC. 2007. Network properties of an epiphyte metacommunity. JOURNAL OF ECOLOGY	Terrestrial	0	1	2	0	0	NA	NA	NA	Host trees are considered to represent the scale of local epiphyte communities
Richter-Boix, A; Llorente, GA; Montori, A. 2007. Structure and dynamics of an amphibian metacommunity in two regions. JOURNAL OF ANIMAL ECOLOGY	Freshwater	0	1	0	0	0	NA	NA	NA	Ponds are considered to represent the scale of local amphibian communities
Stevens, RD; Lopez-Gonzalez, C; Presley, SJ. 2007. Geographical ecology of Paraguayan bats: spatial integration and metacommunity structure of interacting assemblages. JOURNAL OF ANIMAL ECOLOGY	Terrestrial	1	NA	NA	NA	NA	1	0	0	Sampling sites across a mosaic of different habitat types are considered to represent the scale of local bat communities
Werner, EE; Yurewicz, KL; Skelly, DK; Relyea, RA. 2007. Turnover in an amphibian metacommunity: the role of local and regional factors. OIKOS	Freshwater	0	1	0	0	0	NA	NA	NA	Small ponds and large marshes are considered to represent the scale of local amphibian communities
Virtanen, R; Oksanen, J. 2007. The effects of habitat connectivity on cryptogam richness in boulder metacommunity. BIOLOGICAL CONSERVATION	Terrestrial	0	1	0	0	0	NA	NA	NA	Boulders are considered to represent the scale of local criptogam communities
Cronin, JT. 2007. Shared parasitoids in a metacommunity: Indirect interactions inhibit herbivore membership in local communities. ECOLOGY	Terrestrial	0	1	0	0	0	NA	NA	NA	Ponds are considered to represent the scale of specialized grasshoppers- *S. festucacea* communities
Vandvik, V; Goldberg, DE. 2006. Sources of diversity in a grassland metacommunity: Quantifying the contribution of dispersal to species richness. AMERICAN NATURALIST	Terrestrial	1	NA	NA	NA	NA	1	0	0	Sampling sites are considered to represent the scale of the local plant communities
Manier, MK; Arnold, SJ. 2006. Ecological correlates of population genetic structure: a comparative approach using a vertebrate metacommunity. PROCEEDINGS OF THE ROYAL SOCIETY B-BIOLOGICAL SCIENCES	Freshwater	1	NA	NA	NA	NA	1	2	2	The grouping of sites into local populations (local communities) varies across species, and a local population (local communities) may be composed of multiple sites. This is done through genetic analyses
Ellis, AM; Lounibos, LP; Holyoak, M. 2006. Evaluating the long-term metacommunity dynamics of tree hole mosquitoes. ECOLOGY	Freshwater	0	1	0	1	1	NA	NA	NA	Tree water holes are considered to represent the scale of local mosquito communities

Appendix S2. Individual Based Model

S2.1 Technical details of the simulation model

We simulated metacommunities using a continuous space-time spatiotemporal point process (Cornell et al. 2019). The species follow a consumer-resource dynamics, where the individuals' births, deaths and movements are locally governed by top-hat kernels, which define a constant rate per unit area within a specified radius. See Supporting Figure S1 for a graphical model description and Supporting Table S2 for the numerical values of the parameters.

Resource generation and landscape scenarios. We defined $P = 6$ distinct patch types, each producing $L = 2$ resource types, resulting in a total of $R = 12$ resource types. We simulate three landscape scenarios, characterized by the spatial configuration of the resource particles. In the (1) continuous homogeneous and (2) continuous heterogeneous landscape scenarios, the patches are randomly distributed with an expected density of 0.1 patches per unit area. In the (3) patchy landscape scenario, path clusters are generated around randomly distributed seed points also at a density of 0.1. For each seed, the location of all patch types is independently drawn from a normal distribution centered at the seed, with a standard deviation of 0.15. Across all landscape scenarios, the habitat patches uniformly produce resources at a constant rate ψ per unit area within a radius p. Unless consumed, resources disappear at rate γ.

Consumers dynamics and dispersal scenarios. We generated a total of 20 consumer species, comprising of 12 specialists, 6 habitat specialists and 2 generalists. Each specialist species consumes a different resource type, each habitat specialist consumes both resources from a distinct habitat type, and generalists consume all resource types. Consumer individuals can be in 2 states: hungry and resource satiated. Resource consumption occurs at rate λ within radius c of a resource particle. When a resource-satiated individual consumes a resource, it may give birth to a hungry individual at a rate of φ, which immediately disperses up to a distance d. Resource satiated individuals change into resource deprived state at a constant rate β. If a hungry individual consumes a resource particle, it becomes resource satiated. Otherwise, it eventually dies at a rate μ.

Dispersal occurs through a jump process, where resource deprived individuals move at a rate δ to a new location. The maximum distance of each jump is determined by parameter d. There are three dispersal scenarios: (1) all species have short dispersal distance; (2) all species have long dispersal distance; (3) half of the species have short dispersal distance and the other half long dispersal distance. For each consumer species, we assume an immigration rate of ι.

Infection dynamics. We extend the consumer-resource model by simulating parasite infection dynamics on the consumers. We focus on the continuous heterogeneous landscape scenario with mixed dispersal. Parasite dynamics are simulated indirectly by introducing new infected states for consumers. Therefore, each consumer can be both hungry or satiated and healthy or infected. We model six parasite species, assuming they are not habitat-specific, so that each parasite can infect multiple consumer species that specialize on different resources. Each parasite species specializes on two specialist consumers, one habitat specialist and one

generalist. Consequently, generalist consumers are susceptible of infection by three parasite species. Infection between infected and healthy consumer individuals happens at rate α, up to distance a. Once infected, consumer individuals have double the baseline death rate.

A

Process	Before	After	Rate
**Consumer dynamics**			
Consumes and gives birth	◆ ●	◆ ◇	$\Theta\ \lambda c(x_2 - x_3)\varphi f(x_1 - x_2)$
Becomes hungry	◆	◇	β
Consumes resource and becomes satiated	◇ ●	◆	$\Theta\ \lambda c(x_1 - x_2)$
Immigrates		◇	μ
Moves	◇	◇	$\delta d\ (x_1 - x_2)$
**Infection dynamics**			
Infects	◆ ◇	◆ ◆	$\alpha a(x_2 - x_3)\ (x_1 - x_2)$
**Resource dynamics**			
Resource appears		●	$\psi p(x_1 - x_2)$
Resource disappears	●		γ

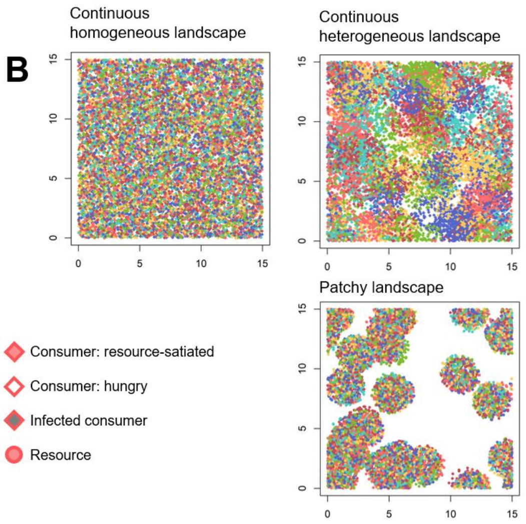

Supporting Figure S1. Illustration of the agent-based model used to simulate resource-consumer dynamics. (A) A graphical model description listing the processes used to model the dynamics of the resources and the consumers. (B) An illustration of resource distributions resulting in the three different model parameterizations that we applied to incorporate qualitatively different metacommunity scenarios.

Supporting Table S2: Parametrization of the model.

Parameter	Description	value
U	Width and height of the landscape	15
P	Number of patch types	6
L	Number of resource types per patch	2
R	Number of resource types	12
$S_{specialist}$	Number of specialist consumer species	R
$S_{partial}$	Number of partial specialist consumer species	P
$S_{generalist}$	Number of generalist consumer species	2
$S_{parasites}$	Number of parasites	6
p	Maximum distance for resource production of a patch	1.5 (patchy and continuous heterogeneous), $U/2$ (continuous homogeneous)
ψ	Resource production rate of a patch	0.5
γ	Resource death rate	0.01
c	Maximum distance for resource consumption	1
λ	Rate of resource consumption	$7.5 \cdot \theta$ (if resource type is within the species niche), otherwise 0
θ	Species-specific weighting of consumption rates to promote regional coexistence	5 (specialists), $\frac{0.225}{L}$ (partial specialists, $\frac{0.225}{2R}$ (generalists)
φ	Consumer birth rate	2.5
β	Transition rate from satiated to hungry state	0.55
μ	Death rate of hungry individuals	0.55 if healthy, 1.1 if infected
δ	Movement rate of hungry individuals	1
d	Maximum movement distance for hungry individuals	0.75 (short dispersal); 1.5 (long dispersal); 0.75 for even-indexed species and 1.5 for odd-indexed species (mixed dispersal).
ι	Immigration rate of consumer species	0.05 if healthy, 0.01 if infected
α	Infection rate	50 for susceptible resource and habitat specialists, 16.6 for susceptible generalist
a	Infection distance	1

S2.2 Convergence criteria

We evaluated the convergence of the definitions by calculating the split-half correlations of the pairwise p_{ij} values, averaged across replicates within each scenario. For the space-use and interaction-based definitions, a sample size of 7,500 yielded Pearson correlation coefficients of $r > 0.99$, indicating convergence between data halves. In contrast, the influence-based definition required 534,00 replicate simulations to achieve an average Pearson coefficient of $r = 0.77$ (sd=0.096). Visual inspection of the correlation plot revealed that most variability occurred around 0 influence values, whereas intermediate and higher influence values better aligned with the identity line. To reduce this noise, we set to zero any values for which their

lower 95% Confidence Interval of the mean overlapped 0. After this post processing, the correlation improved to $r > 0.97$, indicating convergence.

Another indicator of convergence was the cross-modularity values calculated between two independent splits of the replicates, within each definition and across all scenarios. In all cases, the cross-modularity values were >0.9, indicating that each split consistently reached the same internal community structure and was partitioned into the same local communities.

S2.3 Measures of cross-modularity

Given two graphs A and B with the same set of nodes but potentially differing in their edge structure, we define the cross modularity Q_{AB} as a directional similarity score to evaluate the extent to which the community structure derived from graph B aligns with the modular organization of graph A (or vice versa). Specifically, we define cross modularity Q_{AB} as

$$Q_{AB} = \frac{Q(M_A, P_B)}{Q(M_A, P_A)}$$

The modularity function $Q(M, P)$ measures the extent to which partition P captures the modular structure of the graph defined by M, where higher values indicate a higher degree of compartmentalization of the network relative to a null model. That is, strong intra-community connectivities and weak inter-community connectivities. The numerator $Q(M_A, P_B)$ thus quantifies how well the partition independently derived from graph B captures the modularity structure from graph A. The denominator $Q(M_A, P_A)$ provides a normalization factor by representing the maximal modularity achievable by A itself. Therefore $Q_{AB} \epsilon [0,1]$. Values closer to 1 indicate that P_B performs nearly as well as P_A in defining the community structure of graph A, whereas values closer to 0 suggest that the modularity in the connectivities of A does not align well with P_B.

We note that Q_{AB} may not necessarily be equal to Q_{BA}. If both values are high, it indicates high similarity and therefore equivalent community structures. A scenario in which $Q_{AB} \gg Q_{BA}$ (or vice versa) indicates that one graph's community structure generalizes well to the other graph but not the other way around, suggesting topological asymmetries between graphs.

An alternative cross modularity metric, which also captures directionality, was introduced by Diez et al. (2015):

$$Q_{AB} = \sqrt[3]{\bar{S}(P_A, P_B) \times Q(M_A, P_A) \times Q(M_A, P_B)},$$

where $\bar{S}(P_A, P_B)$ is the mean Sørensen similarity between the partitions P_A and P_B across all modules. Although this metric was originally defined to identify an optimal partition shared by two definitions (A and B), it can also be used to assess the alignment between two alternative community structures. As shown by comparison of Fig. 5 of the main text and Supporting Fig.

S2, this alternative measure of cross-modularity yields qualitatively similar results to the measure used in the main manuscript.

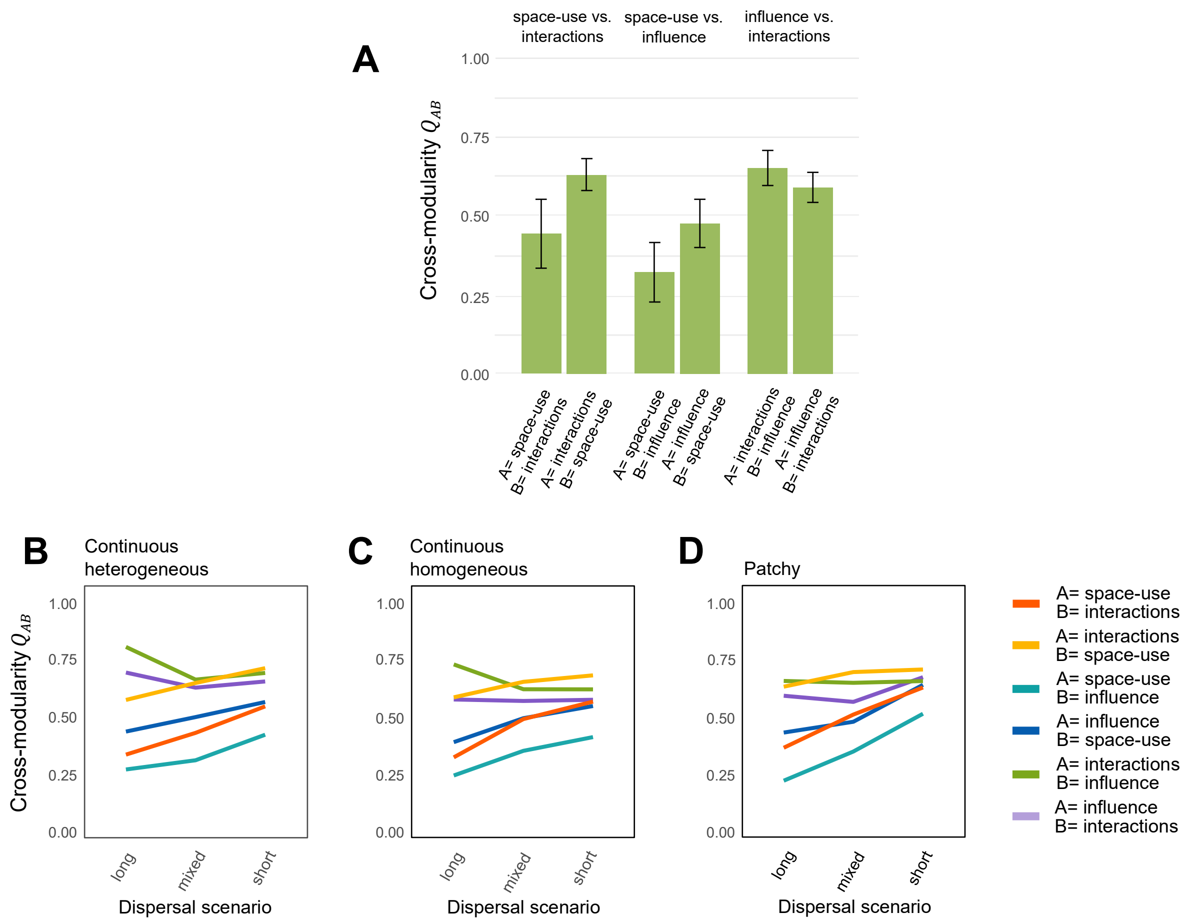

Supporting Figure S2. Cross-modularities of local communities delineated from the agent-based simulations. This figure shows the same information as the panels BCDE in the Fig. 5 of the main article, by assuming the alternative cross-modularity measure proposed by Diez et al. (2015). Panel B shows the mean (the green bar) and one standard error (the black error bars) cross-modularities over the simulated scenarios. Panels EDF show how the cross-modularity values depend on the simulated scenario, the panels referring to landscape types and the x-axes to dispersal scenarios.

S2.3 Illustrations of metacommunity snapshots

Supporting Figures S3-S5 illustrate local communities delineated from different metacommunity scenarios for the consumer resource model, using the space used-based definition (Fig. S3), interaction-based definition (Fig. S4) and influence-based definition (Fig. S5).

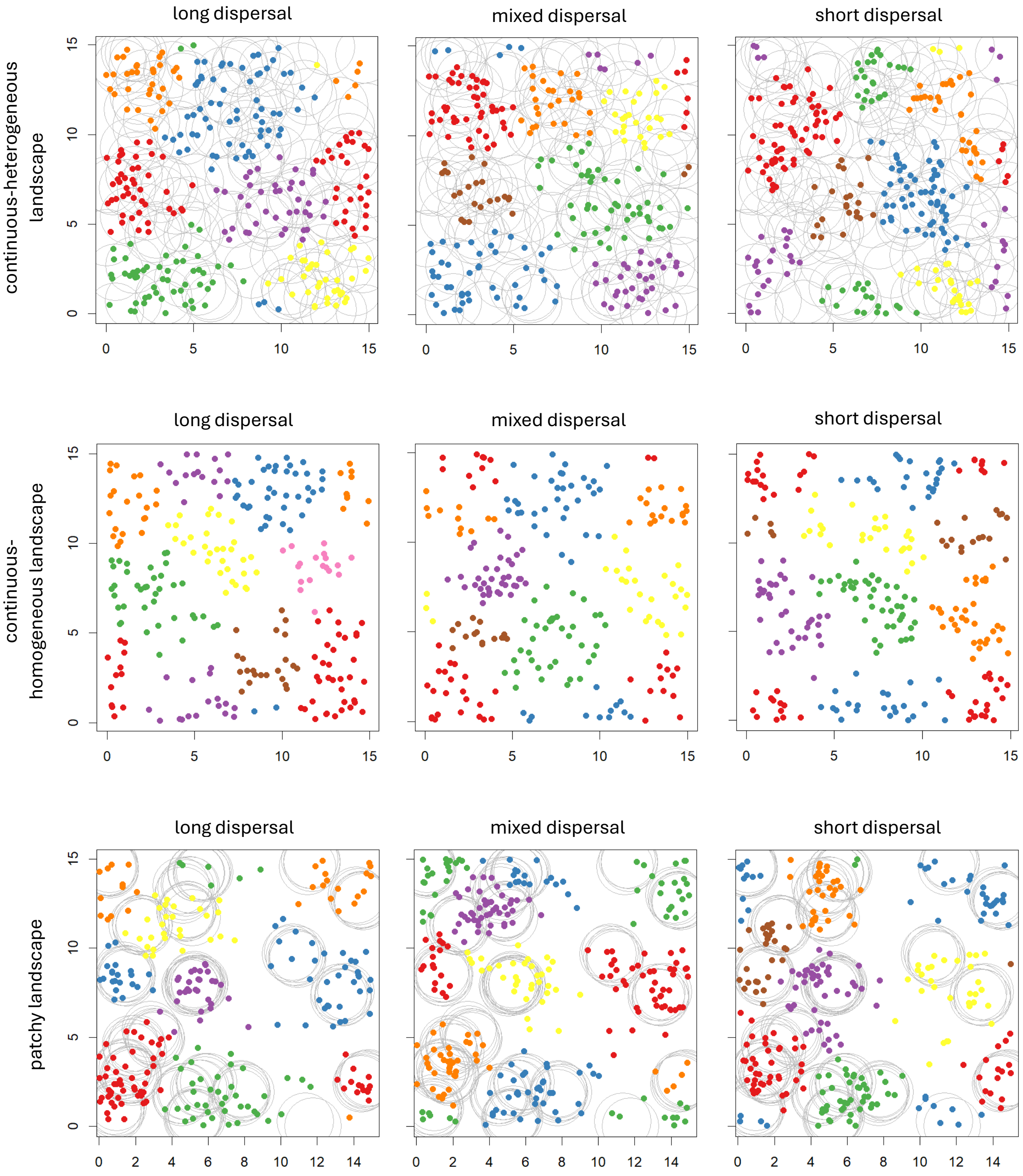

Supporting Figure S3. Snapshots of the local communities as defined by space-use overlap. Large grey circumferences represent resource-generating patches. Other symbols and colors represent individuals that belong to the same local community.

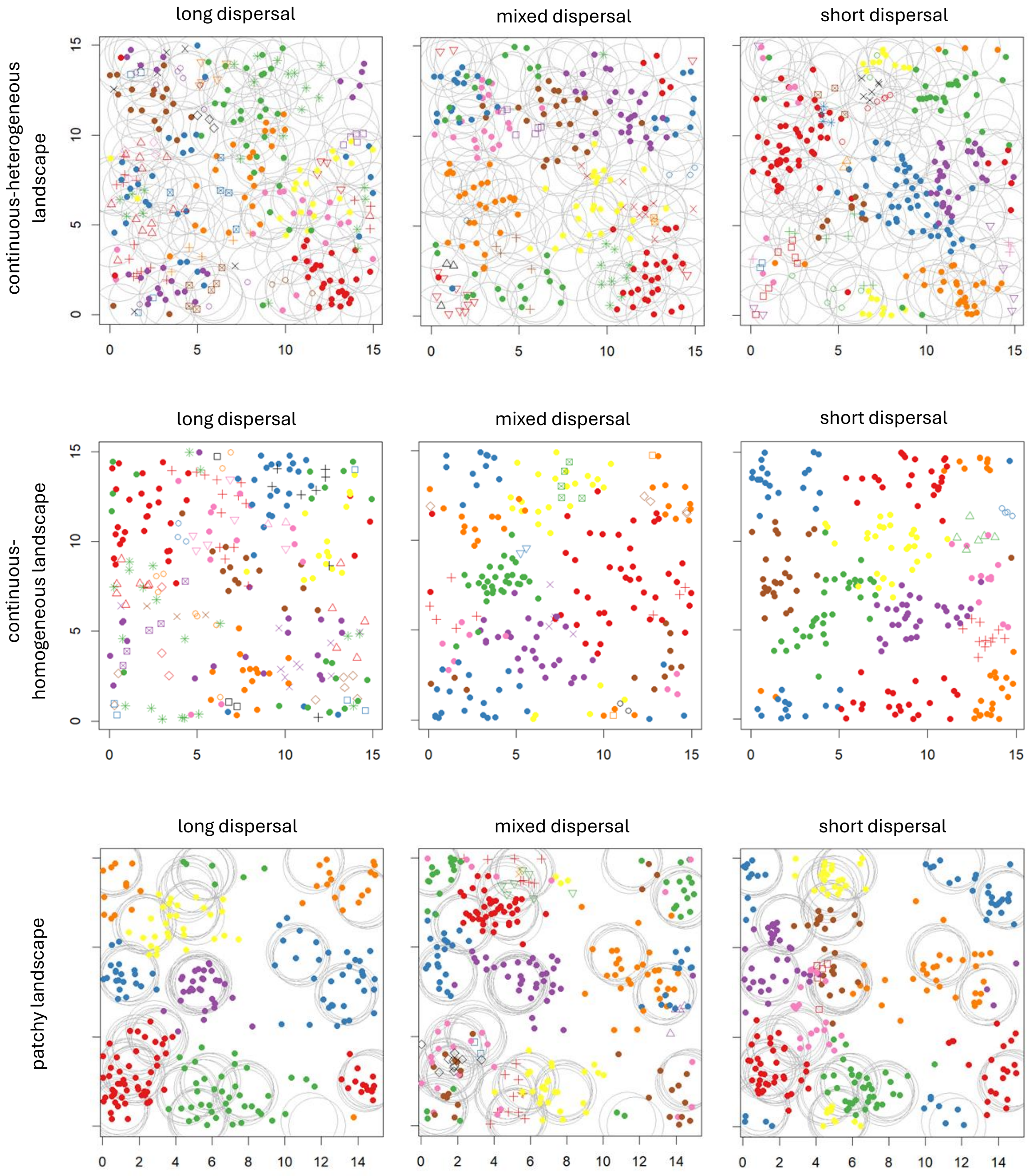

Supporting Figure S4. Snapshots of the local communities as defined by direct interactions. Large grey circumferences represent resource-generating patches. Other symbols and colors represent individuals that belong to the same local community.

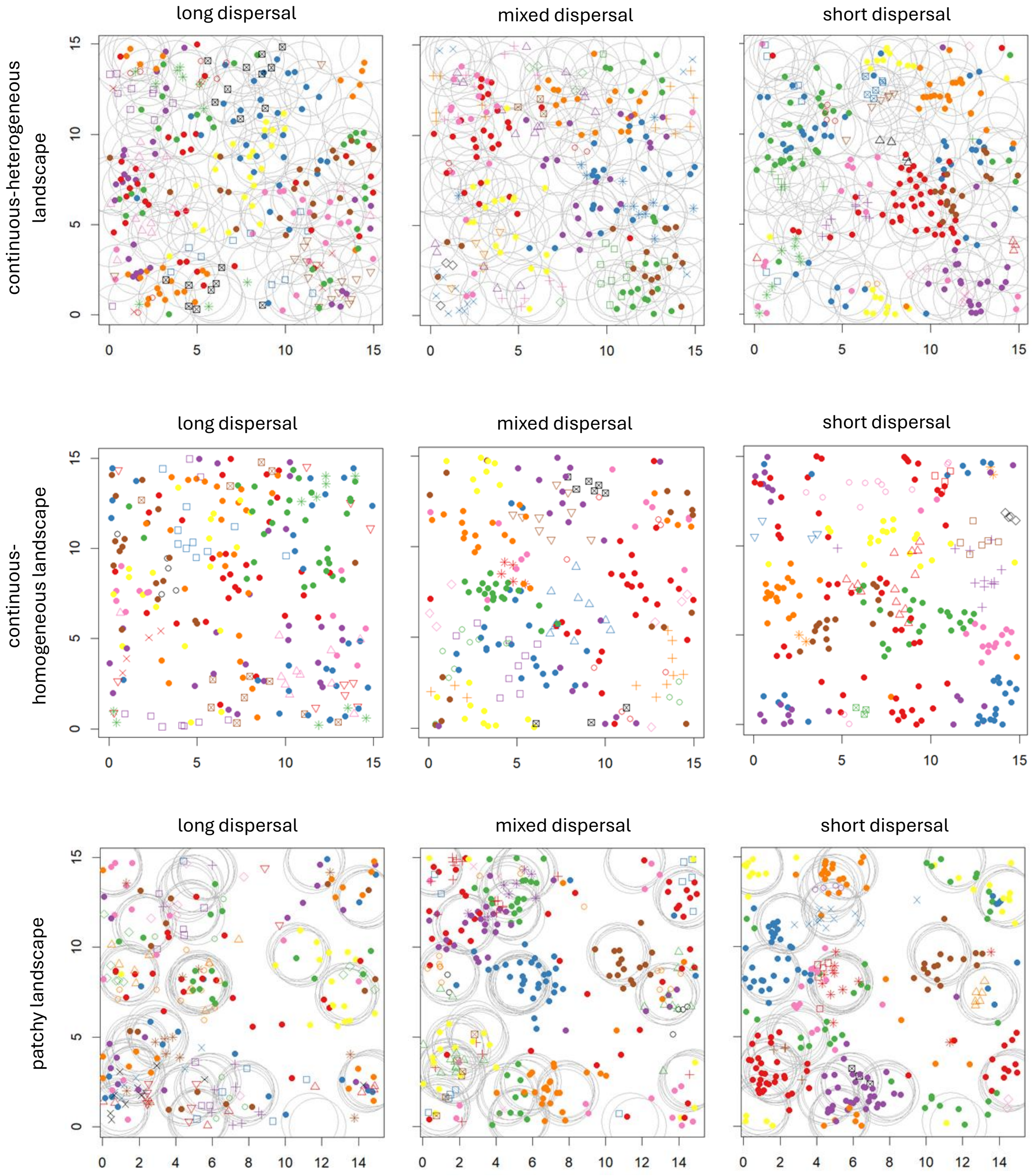

Supporting Figure S5. Snapshots of the local communities as defined by reciprocal fitness influences. Large grey circumferences represent resource-generating patches. Other symbols and colors represent individuals that belong to the same local community.

References

Cornell, S.J., Suprunenko, Y.F., Finkelshtein, D., Somervuo, P. & Ovaskainen, O. (2019). A unified framework for analysis of individual-based models in ecology and beyond. Nat Commun, 10, 4716.

Diez, I., Bonifazi, P., Escudero, I., Mateos, B., Muñoz, M.A., Stramaglia, S., et al. (2015). A novel brain partition highlights the modular skeleton shared by structure and function. Sci Rep, 5, 10532.
